# Polygenic risk scores improve CAD risk prediction in individuals at borderline and intermediate clinical risk

**DOI:** 10.1038/s44325-025-00049-7

**Published:** 2025-05-01

**Authors:** Dariusz Ratman, Placede Tshiaba, Michael Levin, Jiayi Sun, Tate Tunstall, Robert Maier, Premal Shah, Matthew Rabinowitz, Daniel J. Rader, Akash Kumar, Kate Im

**Affiliations:** 1 MyOme Inc, Menlo Park, CA USA; 2 Department of Medicine, University of Pennsylvania, Philadelphia, PA USA; 3 Natera, Inc., San Carlos, CA USA

**Keywords:** Cardiovascular diseases, Atherosclerosis

## Abstract

Polygenic risk scores (PRSs) can improve clinical risk tools for coronary artery disease (CAD). This study assessed a risk model integrating PRS across populations, focusing on individuals with borderline/intermediate clinical risk. We developed ancestry-specific ensemble models combining multi-ancestry PRSs for CAD and type 2 diabetes. The cross-ancestry PRS (caPRS) was integrated with the Pooled Cohort Equations (PCE) to derive the cross-ancestry Integrated Risk Score (caIRS), estimating 10-year CAD risk. The caIRS outperformed the PCE across four cohorts, including UK Biobank and Penn Medicine Biobank, with significant improvements for Hispanic and South Asian individuals. For those at borderline/intermediate PCE risk (5–20%), the caIRS reclassified between 7.0% and 10.7% into the high-risk group, which had higher CAD incidence and hazard ratios ranging from 3.20 to 3.84. The CAD caIRS, combining genetic and clinical factors, enhances high-risk CAD identification across diverse populations, potentially improving treatment guidance.

## Introduction

Coronary artery disease (CAD) is the leading cause of death in the United States^1^. An estimated 20.5 million Americans 20 years of age or older have prevalent CAD^2^. Development of CAD is influenced by several factors, including age, sex, genetics, lifestyle, and comorbidities^3^. However, CAD is preventable, making it a focus of public health efforts to reduce risk through lifestyle therapies and clinical intervention^3^.

The American College of Cardiology/American Heart Association (ACC/AHA) Task Force on Clinical Practice Guidelines currently recommends using the Pooled Cohort Equations (PCE) Atherosclerotic Cardiovascular Disease (ASCVD) risk tool to estimate 10-year risk of a first ASCVD event and to guide decisions on preventive interventions in asymptomatic adults 40–75 years of age^4,5^. The PCE model includes a limited number of established cardiovascular risk factors: age, race, sex, systolic blood pressure, total cholesterol level, high-density lipoprotein cholesterol (HDL-C) level, diabetes status, and smoking status^4^. However, it does not take into account family history or genetic risk and only has two categories of race, Black/African-American and White^4^. It is also limited by its focus on 10-year risk and does not include individuals younger than 40, both of which make it less useful in younger adults.

The calibration of the original PCE model has been demonstrated to vary depending on the target cohort and its characteristics^6,7^. As a consequence, current guidelines recommend considering additional risk-enhancing factors to guide preventive interventions in case of borderline (5-7.5%) and intermediate PCE risk (7.5–20%)^5^. Polygenic risk scores (PRSs), which aggregate information about genetic liability of disease across thousands or millions of genetic variants, have the potential to improve the accuracy of clinical risk prediction tools such as the PCE^8^. Recent studies integrating a CAD PRS with traditional risk factors have shown improved risk prediction for CAD^9,10^, and PRSs have been recognized as potential risk-enhancing factors for CAD risk prediction in a recent AHA Scientific Statement^11^. Nevertheless, generalizability across cohorts and populations remains a major challenge for CAD PRS models. A major factor limiting generalizability has been the paucity of large genetic studies among individuals with diverse genetic backgrounds, and most studies to date have largely validated their models in non-US based populations.

We sought to enhance the performance of CAD risk prediction across diverse populations by constructing a cross-ancestry Integrated Risk Score (caIRS) model. This model combines established clinical risk factors with a cross-ancestry PRS (caPRS) to account for population differences often overlooked in traditional PRS models. The caPRS methodology builds on the previously described model for Breast Cancer risk prediction^12^, which leverages continuous ancestry estimates and population-specific effect size estimates to better tailor risk prediction for genetically diverse populations and further improves it by replacing single PRS models with optimized PRS ensemble scores. The caIRS provides a unified framework that integrates genetic predisposition and traditional clinical predictors. We evaluated the caPRS and caIRS using 4 independent validation cohorts, including a contemporary US-based cohort from the Penn Medicine Biobank (PMBB). We specifically assessed the utility of the caIRS as a screening tool to identify high-risk individuals who have uncertain risk based on traditional risk assessments for CAD and may benefit from early intervention.

## Results

We validated the performance of the CAD caPRS and caIRS in prospectively predicting the 10-year risk of incident CAD using 4 independent, multi-ancestry validation cohorts and compared the predictive performance of the caIRS to that of the PCE model. The corresponding validation cohort characteristics are presented in Table 1.

**Table 1:** Characteristics of the validation cohorts by sex and self-reported ethnicity

Self-reported ethnicity	Total Count	Men		Women		Age^a^	Follow up^a^	Total Cholesterol^a^	HDL Cholesterol^a^	Systolic Blood Pressure^a^	Diabetes	Current Smoker
		Cases	Noncases	Cases	Noncases							
ARIC												
Black/African American	2559	81 (8.2%)	907 (91.8%)	86 (5.5%)	1485 (94.5%)	53.0 (10.0)	23.0 (14.0)	211.0 (58.0)	34.7 (14.4)	126.0 (26.0)	435 (17.0%)	768 (30.0%)
White/Caucasian	8449	441 (11.4%)	3442 (88.6%)	166 (3.6%)	4400 (96.4%)	54.0 (10.0)	25.0 (12.0)	211.0 (51.0)	32.1 (13.8)	117.0 (22.0)	515 (6.1%)	2137 (25.3%)
All	11008	522 (10.7%)	4349 (89.3%)	252 (4.1%)	5885 (95.9%)	54.0 (10.0)	25.0 (13.0)	211.0 (53.0)	32.7 (14.3)	118.0 (23.0)	950 (8.6%)	2905 (26.4%)
MESA												
Black/African American	1067	44 (8.7%)	463 (91.3%)	17 (3%)	543 (97%)	60.0 (17.0)	10.1 (0.9)	189.0 (46.5)	50.0 (19.5)	128.5 (28.2)	141 (13.2%)	223 (20.9%)
Hispanic	828	38 (8.9%)	388 (91.1%)	10 (2.5%)	392 (97.5%)	59.0 (16.0)	10.2 (1.1)	197.0 (45.0)	44.0 (15.0)	121.5 (28.5)	119 (14.4%)	127 (15.3%)
East Asian/Asian American	526	12 (4.4%)	259 (95.6%)	3 (1.2%)	252 (98.8%)	61.0 (16.0)	10.3 (1.0)	192.0 (35.8)	47.0 (16.0)	119.0 (27.9)	43 (8.2%)	38 (7.2%)
White/Caucasian	1741	76 (9.2%)	751 (90.8%)	40 (4.4%)	874 (95.6%)	61.0 (16.0)	10.4 (1.1)	198.0 (43.0)	50.0 (21.0)	119.5 (26.0)	83 (4.8%)	207 (11.9%)
All	4162	170 (8.4%)	1861 (91.6%)	70 (3.3%)	2061 (96.7%)	60.0 (17.0)	10.2 (1.0)	195.0 (44.0)	48.0 (19.0)	122.0 (28.0)	386 (9.3%)	595 (14.3%)
UKB												
Black / Black British	1842	15 (1.9%)	758 (98.1%)	8 (0.7%)	1061 (99.3%)	49.0 (11.0)	11.9 (1.2)	204.5 (52.7)	53.9 (17.4)	135.0 (25.0)	282 (13.4%)	303 (14.4%)
East Asian/Asian American	409	3 (2%)	144 (98%)	3 (1.1%)	259 (98.9%)	52.0 (13.0)	12.2 (1.3)	214.5 (44.7)	55.5 (19.9)	128.5 (24.0)	31 (7.6%)	28 (6.8%)
White/Caucasian	113904	2070 (4.2%)	47300 (95.8%)	825 (1.3%)	63709 (98.7%)	57.0 (13.0)	12.4 (1.5)	226.8 (54.1)	55.6 (19.6)	135.5 (24.5)	5120 (4.5%)	11863 (10.4%)
Other	2669	38 (3.2%)	1135 (96.8%)	19 (1.3%)	1477 (98.7%)	50.0 (13.0)	12.1 (1.4)	216.7 (53.3)	52.0 (19.6)	131.0 (23.5)	243 (8.8%)	425 (15.4%)
(South) Asian or Asian British	1766	52 (5.9%)	824 (94.1%)	17 (1.9%)	873 (98.1%)	51.0 (13.0)	12.0 (1.2)	212.0 (49.8)	47.6 (16.0)	132.8 (24.0)	298 (16.9%)	159 (9.0%)
All	120590	2178 (4.2%)	50161 (95.8%)	872 (1.3%)	67379 (98.7%)	56.0 (13.0)	12.4 (1.5)	226.1 (54.1)	55.3 (19.6)	135.5 (24.5)	5920 (4.9%)	12737 (10.6%)
PMBB												
Black/African American	2858	94 (8.4%)	1029 (91.6%)	64 (3.7%)	1671 (96.3%)	55.0 (15.0)	5.3 (4.4)	178.0 (45.0)	51.0 (19.0)	129.0 (16.0)	418 (14.6%)	474 (16.6%)
East Asian/Asian American	221	12 (10.9%)	98 (89.1%)	5 (4.5%)	106 (95.5%)	52.0 (15.0)	4.8 (5.8)	184.0 (48.0)	51.0 (20.0)	120.0 (21.0)	30 (13.6%)	9 (4.1%)
Hispanic	803	60 (13.3%)	391 (86.7%)	18 (5.1%)	334 (94.9%)	57.0 (16.0)	4.4 (5.9)	184.0 (46.0)	51.0 (19.0)	125.0 (16.0)	78 (9.7%)	59 (7.3%)
White/Caucasian	10300	690 (13.1%)	4590 (86.9%)	215 (4.3%)	4805 (95.7%)	59.0 (16.0)	4.9 (5.7)	185.0 (48.0)	52.0 (22.0)	124.0 (17.0)	947 (9.2%)	713 (6.9%)
All	14182	856 (12.3%)	6108 (87.7%)	302 (4.2%)	6916 (95.8%)	58.0 (16.0)	5.0 (5.5)	184.0 (47.0)	52.0 (22.0)	125.0 (17.0)	1473 (10.4%)	1255 (8.8%)

Cohort labels: *ARIC* - Atherosclerosis Risk In Communities, *MESA* - Multiethnic Study of Atherosclerosis, *UKB* - UK Biobank, *PMBB* - Penn Medicine Biobank.

^a^median(IQR).

### Predictive power of the caPRS in 10-year incident CAD risk prediction across ethnicities

To evaluate the predictive performance of the caPRS in estimating the 10-year risk of incident CAD, we used a Cox PH model, adjusting for baseline age and sex within each validation cohort and distinct self-reported ethnicity groups, analyzed independently.

**Fig. 1:**
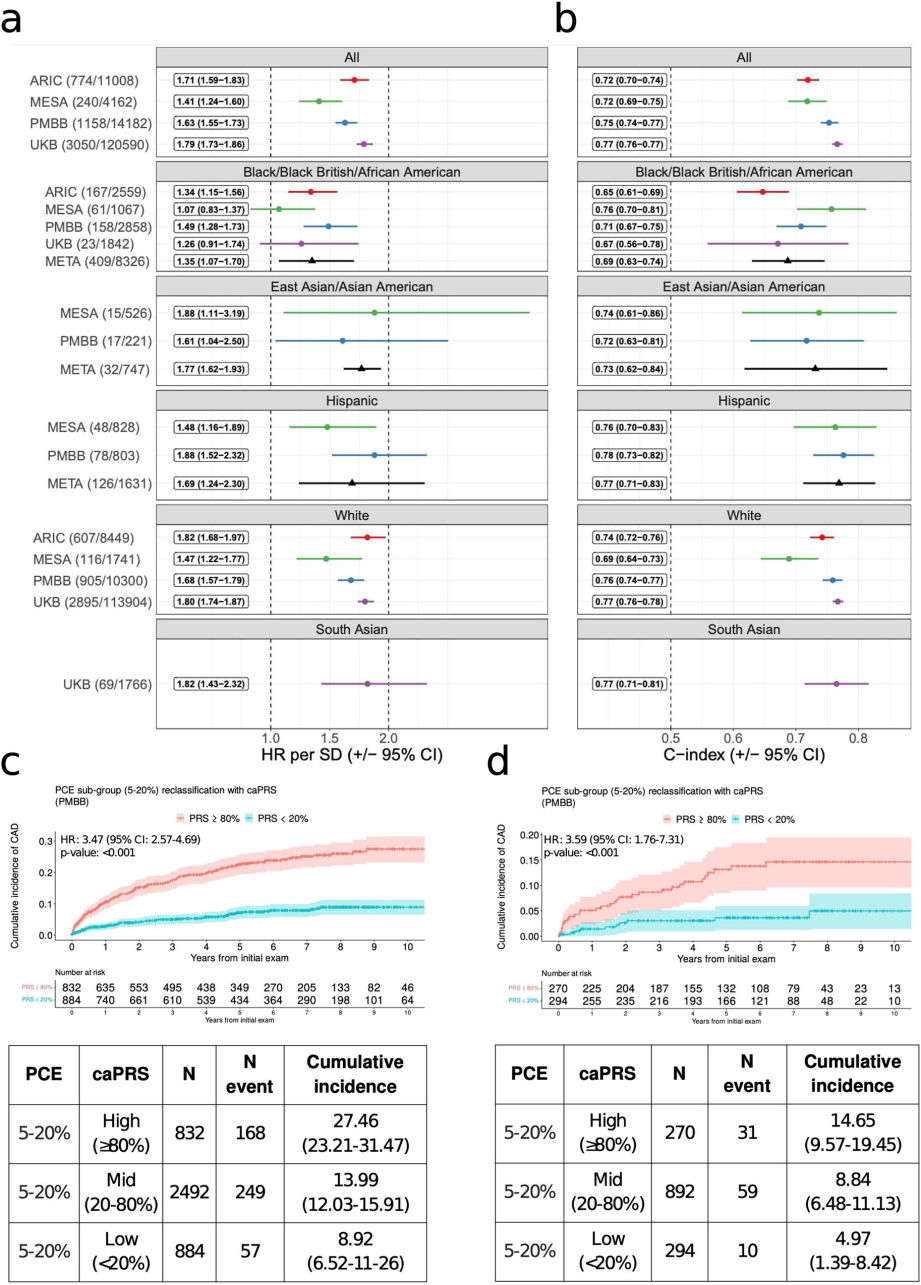
Cox PH model results for the association between caPRS and 10-year CAD incidence across validation cohorts and self-reported ethnicity groups. **a** caPRS Hazard Ratio (HR) adjusted for age and sex and **b** the corresponding C-index of the Cox PH model. Values on the left correspond to the plotted point estimates and their 95% CI. Cohort labels include count of incident cases and the total. META corresponds to the meta analysis of underrepresented ethnicities. **c**, **d** PRS stratified cumulative incidence of CAD among the borderline/intermediate (PCE) risk group for the White and African-American populations, respectively.

The association between caPRS and 10-year CAD incidence was statistically significant at the 0.05 level across all 4 validation cohorts. The overall HR per SD ranged from 1.41 to 1.79 with a corresponding C-index from 0.72 to 0.77 (Fig. 1a and b). The caPRS was significantly associated with incident CAD across all (self-reported) ethnic subgroups. The strongest associations observed in Hispanic (HR per SD: 1.69; 95% CI, 1.24–2.30), East Asian (Asian American) individuals (HR per SD: 1.77; 95% CI, 1.62–1.93) and South Asian individuals (HR per SD: UKB, 1.82; 95% CI, 1.43–2.32) were comparable in magnitude to the effects observed among White population (HR per SD ranging from 1.47 to 1.82). However, the association was relatively weaker in the Black/African American individuals, with an HR per SD of 1.35 (95% CI, 1.07–1.80).

We additionally assessed PRS-based risk stratification among individuals at uncertain (borderline/intermediate) clinical risk (PCE) (Fig. 1c and d). Within the White population, high caPRS (top 20%) was associated with a 10-year CAD incidence of 27.5% (95% CI, 23.2–31.5) as compared to 14.0% (95% CI, 12.0–15.9) and 8.9% (95% CI, 6.5–11.2) for those with average (mid 40–60%) and low (bottom 20%) PRS. While in the case of Black/African-American population, high PRS was associated with a CAD incidence of 14.6% (95% CI, 9.6–19.4) compared with 8.8 (95% CI, 6.5–11.1) and 5.0% (95% CI, 1.4–8.4) for those with average and low PRS.

We observe a similar stratification of individuals at <5% and ≥20% clinical risk with individuals in the top 20% of caPRS having a higher incidence of disease overall in validation cohorts (Supplementary Figs. 1 and 2).

In head-to-head comparison, the caPRS model demonstrated improved performance compared to the recently developed multiancestry GPSMult^9^ model (and other models from PGS catalog), specifically within East Asian/Asian American and Black/African-American populations, with equivalent performance to GPSMult in pooled analysis across all populations (Supplementary Fig. 3).

### Evaluation of the caIRS in comparison to PCE

We developed the caIRS model by combining the caPRS with the PCE, which serves as the current standard for assessing ASCVD risk within the US. We compared the performance of the caIRS to the PCE alone in the identification of individuals at high risk of developing CAD over a 10-year period.

The caIRS model consistently outperformed the baseline PCE across all validation cohorts, exhibiting improvements in discrimination as reflected by the C-index. The improvements ranged from 1.5 percentage points in MESA to 3 percentage points in UKB (Table 2). When using a fixed classification threshold of 20%, corresponding to ACA/AHA “high risk” threshold^5^, we observed a consistent increase in sensitivity, PPV, and NPV across all validation cohorts with a marginal decrease in specificity, except for in MESA (Table 2). This improvement was further corroborated by the positive and significant NRI values, which ranged from 5.9% (95% CI, 1.2–11.3) in MESA to 9.8% (95% CI, 8.3–11.5) in UKB (Table 2). The caIRS also outperformed the baseline PCE model when using lower classification thresholds of 7.5% and 10%, with the exception of the MESA cohort where the overall NRI did not reach statistical significance (Supplementary Table 1). Those alternative risk thresholds correspond to the recommendation for statin initiation by the US Preventive Services Task Force Recommendation Statement (7.5%)^13^ and European Prevention Guidelines (10%)^14^.

**Table 2:** Comparative performance of the PCE and caIRS across validation cohorts

Model	Cohort	N (cases)	NRI (95% CI)	Sensitivity	Specificity	PPV	NPV	C-index (95% CI)
PCE	ARIC	11008 (774)	–	28.7	92	21.3	94.5	0.758 (0.743–0.773)
caIRS			9.51 (6.44–12.48)	39.7	90.5	24	95.2	0.783 (0.768–0.798)
PCE	MESA	4162 (240)	–	39.6	81.7	11.7	95.7	0.717 (0.689–0.746)
caIRS			5.86 (1.16–11.32)	42.9	84.3	14.3	96	0.732 (0.704–0.761)
PCE	PMBB	14182 (1158)	–	29.4	91.3	23.1	93.6	0.732 (0.719–0.746)
caIRS			9.08 (6.63–11.59)	39	90.8	27.4	94.4	0.761 (0.747–0.774)
PCE	UKB	120590 (3050)	–	25.5	93.8	9.6	98	0.767 (0.759–0.774)
caIRS			9.84 (8.32–11.47)	37.6	91.5	10.3	98.3	0.797 (0.79–0.804)

The Sensitivity, Specificity, Positive Predictive Value (PPV), Negative Predictive Value (NPV), and Net Reclassification Improvement (NRI) values correspond to the 20% risk classification threshold.

When examining self-reported ethnicity subgroups, the caIRS also surpassed the performance of the PCE model, with the most significant improvements seen among Hispanic (PMBB) and South Asian (UKB) individuals, where the NRI reached 16.2% (95% CI, 6.1–25.8) and 15.0% (95% CI, 6.1–24.2), respectively (Supplementary Table 2).

We also assessed the calibration of both the baseline PCE and caIRS models and found that both tended to overestimate the risk, mainly in the UKB and MESA cohorts, where CAD incidence is relatively lower, and, to a lesser extent in ARIC (Supplementary Fig. 4). The calibration was best in the case of the PMBB cohort where the average predicted risk only slightly deviated from the observed incidence for both models (PCE: 8.4%; caIRS: 8.8%; actual incidence: 8.2%).

### Refining CAD risk stratification in intermediate/borderline risk individuals

Decisions on the initiation and intensity of statin therapy for individuals with a borderline/intermediate PCE risk depend on the presence of one or more risk-enhancing factors, such as family history of ASCVD, metabolic syndrome, and chronic kidney disease^3^. In its recent scientific statement, the AHA/ACC also recognized PRS as a potential risk-enhancing factor^11^.

To understand the utility of the caIRS in refining clinical risk estimates, we tested its performance among the subset of individuals classified as borderline/intermediate risk by the standard PCE. We used the recommended PCE risk threshold of 5% to less than 20% to assign subjects into the borderline/intermediate risk group and applied a 20% threshold to identify individuals who would be considered at high risk when PRS is factored into risk estimation. This 20% risk threshold corresponds to a Class I AHA/ACC recommendation for statin initiation^5^.

**Fig. 2:**
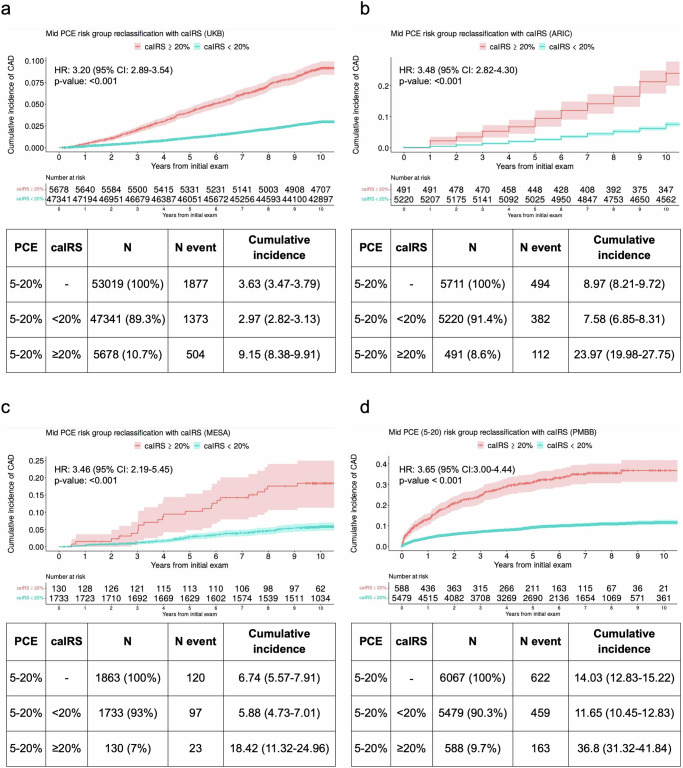
10-year cumulative incidence of CAD among individuals identified as borderline or intermediate risk using PCE and those reclassified into high and low-risk groups by caIRS with the corresponding 10-year cumulative incidence rates (+/− 95% CI), counts of individuals (N) and events (N event) for each group. **a** UKB, **b** ARIC, **c** MESA, **d** PMBB.

Across all validation cohorts, we observed a clear separation in the 10-year cumulative incidence of CAD between the high caIRS (≥20%) group and all others (<20%) (Fig. 2). The largest difference was observed in the PMBB where the 10-year cumulative incidence of CAD in the high caIRS group was 36.8% (95% CI, 31.3–41.8), compared to 11.6% (95% CI, 10.4–12.8) in all others and 14% (95% CI, 12.8–15.2) in the overall borderline/intermediate PCE group. Importantly, the caIRS model identified additional cases which would have been otherwise missed by the PCE alone with sensitivity ranging between 19.0% (MESA) and 26.8% (UKB). In PMBB, where the baseline PCE model demonstrated best calibration, the caIRS was able to reclassify 163 of 622 cases (26%) as high risk.

We observed consistent risk stratification results across all validation cohorts with the HR for 10-year CAD for the high caIRS group compared to all others (classified as borderline/intermediate by the PCE) ranging between 3.20 and 3.84 (Fig. 2).

### Refining CAD risk stratification among Black/African-American population at intermediate/borderline risk

**Fig. 3:**
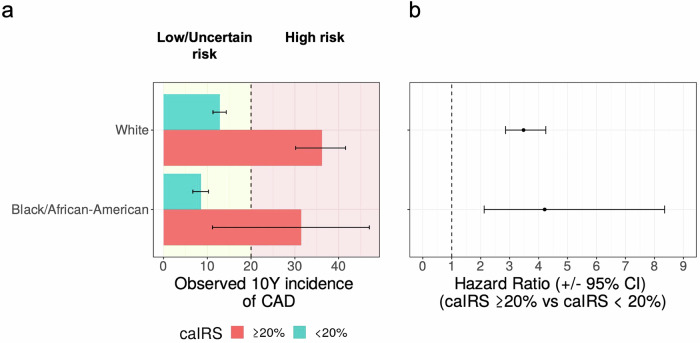
Reclassification of the borderline/intermediate PCE risk group using caIRS among Black/African-American and White individuals in PMBB. **a** Observed 10Y incidence of CAD for high (>=20%) and low caIRS (<20%) and **b** the corresponding hazard ratios for the high vs low caIRS groups.

To assess the reclassification performance across self-reported ethnicity groups, we independently applied the caIRS to Black/African-American and White individuals at the borderline/intermediate PCE risk within the PMBB cohort (Fig. 3). The Black/African-American population reclassified into the high-risk group by the caIRS experienced significantly elevated CAD risk (*P* < 0.001) with the HR between the high-risk caIRS group and all others 4.21 (95% CI, 2.12–8.35) comparable to the White population (HR, 3.48; 95% CI, 2.86–4.25) and the observed 10-year incidence of CAD consistent with the expected high-risk threshold.

## Discussion

We developed and validated an integrated risk score for prediction of CAD in individuals of diverse ancestries by combining a caPRS with the PCE risk estimator, a commonly used tool to predict 10-year risk of a first ASCVD event^4,5^. Unlike prior studies that predominantly focused on European populations, we utilized genetically diverse development cohorts, ensuring broader applicability. To construct the CAD caPRS, we incorporated ensemble PRS into the previously described caPRS development framework^12^. The caIRS further integrates caPRS and clinical risk factors into a unified risk prediction and includes a calibration constant to ensure that the average risk based on caIRS aligns with the average risk predicted by PCE alone for unaffected controls within each decile of the PCE score. Our study builds on prior efforts to improve PRS performance across diverse populations^9,10^ and demonstrates that the caPRS significantly improves risk prediction for CAD in individuals of diverse ancestries beyond traditional risk factors. Through extensive validation using 4 independent cohorts, including the contemporary US-based PMBB, our results add to the growing body of evidence supporting potential utility of PRSs in refining risk estimates for CAD and improving primary prevention efforts.

Current screening paradigms for primary prevention of CAD in the US do not account for genetic risk factors and fail to identify individuals at high polygenic risk^15^, which represents a missed opportunity for preventive interventions. We designed our study with the goal of building a PRS and integrated model applicable across multiple ancestries, including those traditionally underrepresented in GWAS studies. By incorporating multiethnic GWAS summary statistics^16–21^ in PRS ensemble construction and including non-European cohorts (Table 3) in the caPRS and caIRS development, we were able to obtain a model with a strong discrimination capacity across multiple ancestries. This is exemplified by the robust association of the caPRS (HR per SD) with incident CAD in Asian and Hispanic individuals, comparable in magnitude to White individuals. Similarly, we observed notable improvements in classification metrics when comparing the caIRS model with PCE with a NRI of 16.2% (95% CI, 6.1–25.8), 15.0% (95% CI, 6.1–24.2), and 9.8% (95% CI, −13.3–34.0) for Hispanic (PMBB), South Asian (UKB), and East Asian (Asian American, MESA) ethnicities, respectively.

**Table 3:** Characteristics of the development cohorts by sex and genetically inferred ancestry (classified based on similarity to 5 continental superpopulations)

Cohort	Ancestry	Total Count	Men		Women		Age^a^	Total Cholesterol^a^	HDL Cholesterol^a^	Systolic Blood Pressure^a^	Diabetes	Current Smoker
			Cases	Noncases	Cases	Noncases						
Development 1												
HCHS	AMR	2827	94 (8.0%)	1078 (92.0%)	97 (5.9%)	1558 (94.1%)	46.0 (19.0)	197.0 (55.0)	47.0 (16.0)	119.0 (21.0)	498 (17.6%)	568 (20.1%)
JHS	AFR	927	58 (16.5%)	294 (83.5%)	86 (15.0%)	489 (85.0%)	48.0 (13.0)	195.0 (55.0)	48.0 (18.0)	122.9 (20.2)	161 (17.4%)	125 (13.5%)
UKB	AFR	2479	75 (7.2%)	960 (92.8%)	61 (4.2%)	1383 (95.8%)	50.0 (11.0)	203.1 (54.6)	53.9 (18.3)	136.5 (25.0)	375 (15.1%)	272 (11.0%)
	AMR	500	27 (13.5%)	173 (86.5%)	8 (2.7%)	292 (97.3%)	49.0 (12.0)	214.7 (52.4)	54.7 (18.5)	130.5 (24.2)	47 (9.4%)	95 (19.0%)
	EAS	887	22 (8.0%)	253 (92.0%)	9 (1.5%)	603 (98.5%)	51.0 (12.0)	219.5 (55.3)	55.3 (19.8)	128.0 (26.0)	69 (7.8%)	72 (8.1%)
	EUR	158240	12827 (17.8%)	59409 (82.2%)	4015 (4.7%)	81989 (95.3%)	57.0 (13.0)	223.2 (56.6)	54.7 (19.7)	135.5 (25.0)	9868 (6.2%)	16371 (10.3%)
	SAS	1586	254 (30.1%)	591 (69.9%)	47 (6.3%)	694 (93.7%)	53.0 (14.0)	209.0 (59.7)	47.0 (16.3)	132.5 (24.0)	393 (24.8%)	152 (9.6%)
All		167446	13357 (17.6%)	62505 (82.4%)	4323 (4.7%)	87008 (95.3%)	57.0 (14.0)	222.2 (56.9)	54.4 (19.6)	135.0 (25.0)	11411 (6.8%)	17655 (10.5%)
Development 2												
HCHS	AMR	1187	34 (7.3%)	430 (92.7%)	53 (7.3%)	670 (92.7%)	46.0 (19.0)	197.0 (56.0)	48.0 (16.0)	118.0 (22.0)	232 (19.5%)	212 (17.9%)
JHS	AFR	366	23 (16.2%)	119 (83.8%)	30 (13.4%)	194 (86.6%)	48.5 (15.0)	190.0 (52.0)	47.0 (15.3)	121.5 (20.9)	68 (18.6%)	53 (14.5%)
UKB	AFR	1035	29 (6.6%)	413 (93.4%)	25 (4.2%)	568 (95.8%)	50.0 (11.0)	202.2 (51.2)	54.1 (18.7)	135.5 (24.5)	166 (16.0%)	122 (11.8%)
	AMR	230	8 (9.2%)	79 (90.8%)	8 (5.6%)	135 (94.4%)	49.0 (12.0)	205.7 (61.4)	52.3 (18.7)	129.0 (23.5)	21 (9.1%)	43 (18.7%)
	EAS	390	4 (3.3%)	117 (96.7%)	7 (2.6%)	262 (97.4%)	51.0 (12.0)	218.4 (51.4)	55.9 (19.5)	130.5 (25.4)	22 (5.6%)	27 (6.9%)
	EUR	67767	5324 (17.3%)	25457 (82.7%)	1754 (4.7%)	35232 (95.3%)	57.0 (13.0)	223.4 (56.1)	54.6 (19.7)	135.5 (25.0)	4203 (6.2%)	7091 (10.5%)
	SAS	669	90 (26.5%)	250 (73.5%)	22 (6.7%)	307 (93.3%)	52.0 (14.0)	212.1 (54.5)	47.4 (17.2)	132.0 (26.0)	138 (20.6%)	66 (9.9%)
All		71644	5512 (17%)	26865 (83%)	1899 (4.9%)	37106 (95.1%)	57.0 (14.0)	222.3 (56.6)	54.4 (19.6)	135.0 (25.0)	4850 (6.8%)	7614 (10.6%)
Development 3												
CHS	ADMIXED	211	28 (33.3%)	56 (66.7%)	31 (24.4%)	96 (75.6%)	71.0 (6.0)	208.8 (47.0)	55.0 (20.5)	136.0 (26.0)	47 (22.3%)	27 (12.8%)
	AFR	343	42 (32.3%)	88 (67.7%)	57 (26.8%)	156 (73.2%)	71.0 (6.0)	209.0 (44.9)	55.0 (20.0)	140.8 (29.9)	82 (23.9%)	66 (19.2%)
	AMR	29	4 (40%)	6 (60%)	2 (10.5%)	17 (89.5%)	69.0 (4.0)	214.8 (37.2)	51.0 (24.0)	142.8 (27.0)	7 (24.1%)	1 (3.4%)
	EUR	2290	492 (50.5%)	482 (49.5%)	388 (29.5%)	928 (70.5%)	71.0 (6.0)	211.8 (51.0)	50.5 (20.0)	133.0 (27.0)	245 (10.7%)	270 (11.8%)
HCHS	ADMIXED	762	67 (16.1%)	349 (83.9%)	42 (12.1%)	304 (87.9%)	53.0 (12.0)	204.0 (59.0)	46.0 (16.8)	125.0 (24.0)	172 (22.6%)	415 (54.5%)
JHS	ADMIXED	178	9 (12%)	66 (88%)	17 (16.5%)	86 (83.5%)	51.0 (11.8)	197.0 (40.7)	47.0 (17.0)	122.9 (20.0)	27 (15.2%)	27 (15.2%)
UKB	ADMIXED	4929	517 (21%)	1947 (79%)	136 (5.5%)	2329 (94.5%)	51.0 (14.0)	213.8 (57.2)	49.7 (17.9)	131.5 (23.5)	733 (14.9%)	703 (14.3%)
All		8742	1159 (27.9%)	2994 (72.1%)	673 (14.7%)	3916 (85.3%)	59.0 (20.0)	211.4 (54.5)	50.0 (18.4)	131.8 (25.5)	1313 (15.0%)	1509 (17.3%)

Cohort labels: *HCHS* Hispanic Community Health Study, *JHS* Jackson Heart Study, *UKB* UK Biobank, *CHS* Cardiovascular Health Study. Genetic ancestry labels: *AFR* African, *AMR* Admixed American, *EAS* East Asian, *EUR* European, *SAS* South Asian, ADMIXED individuals classified as admixed/other.

^a^median(IQR).

Improved risk assessment for CAD has the potential to substantially impact the use of preventive interventions. Our results demonstrate that the caPRS and integrated model can efficiently refine risk estimates for individuals in the borderline and intermediate PCE risk categories, including Black/African-American population, traditionally underrepresented in genetic research. In the case of PMBB, where both (caIRS and PCE) models were well calibrated, the caIRS reclassified 9.7% of the borderline/intermediate risk subjects into the high-risk group which experienced almost four-fold increase in CAD incidence. By validating the caIRS across diverse cohorts with varied population structures and case definitions, we demonstrated the robustness of our methodology in real-world applications. For example, if all individuals reclassified as high risk (starting with those in the borderline/intermediate clinical risk category) initiated statin use and we assume taking statins results in a 25% reduction in CAD events^22^, then we can extrapolate that 1 additional event over 10 years could be prevented for every 179 individuals screened using the caIRS based on performance in our three US validation cohorts (PMBB, ARIC, MESA). This estimated reduction in events may be even greater as a number of studies reported that patients in the high end of the polygenic risk spectrum experience higher relative and absolute risk reduction from cholesterol-lowering medications, such as statins or PCSK9 inhibitors^23,24^.

Despite improvements in reclassification of Black/African-American individuals at borderline/intermediate clinical risk (when using caPRS and caIRS), the overall performance within this population was attenuated compared to other ethnicities. This likely reflects the reduced availability of GWAS summary statistics and individual-level data for PRS development, which is further compounded by the high genetic diversity of the African population^25,26^. In addition, recent studies demonstrated a continuous decay of PRS performance as the sample’s genetic distance from the training cohort increases^27^. This may further contribute to attenuated performance as the PRS developed in this study relied primarily on GWASs derived from European and Asian populations. Significant performance improvements will require access to larger GWAS studies with better representation of African individuals. The on-going initiatives to improve the diversity in genetics research, such as the All of Us research program^28^, and Million Veterans Program^29^ are expected to contribute to reducing the gap in PRS performance, but existing limitations should be carefully considered as PRSs begin to enter clinical practice.

The results presented here underscore the value of PRS as a risk-enhancing factor for CAD and warrant further prospective validation in a real-world setting. The NHGRI-funded eEMERGE-IV consortium is currently assessing the clinical impact of incorporating PRS for several conditions, including CAD, into a genome-informed risk assessment and delivering this to the EHR with clinical decision support^30^. In addition, we are currently embarking on a prospective clinical study to assess the utility of the CAD caIRS in primary prevention (trial registration number: NCT06542432).

Our study has some limitations. First, this study only examined CAD as an outcome, whereas the PCE was developed to predict the risk of ASCVD, which also includes fatal and nonfatal stroke.

Second, in line with previous studies^6,7^, we observed that the baseline PCE model tended to overestimate the risk to various extents depending on the target cohort. This was especially evident in the case of the UKB cohort, which is generally biased towards healthier individuals and where CAD incidence rates are lower than in US cohorts^31^. Nevertheless, we consider the original PCE model as the most appropriate baseline for comparison as this is the model which is being used for risk assessment in clinical practice in the US. We observed the best calibration in PMBB, which is a contemporary US-based health system. However, this may not be the case across all US populations and in a real-world setting recalibration of the model to a given health-system population could be considered if necessary^6^.

Third, this study did not evaluate use of the caIRS in guiding treatment decisions or improving patient outcomes in a clinical setting. Additional prospective, real-world evidence is needed to support the utility of caIRS in this context.

Fourth, the largest development and validation cohort in this study was UKB. This is not ideal given that most individuals are of European ancestry and there is a lower incidence of CAD compared to the US cohorts. Despite these issues, the caIRS model outperformed the PCE across all validation cohorts. Future studies utilizing more diverse biobanks in the development and validation process should be conducted to further improve performance and generalizability of the caIRS model.

Fifth, the study was restricted to individuals aged 40–79, the age range for which the PCE is intended to be used. However, PRS may be most effective in a prevention setting among a younger population for whom current clinical risk factor-based tools are not well developed or recommended. A recent study showed that the incidence of very early-stage atherosclerosis increased with PRS quintile^32^.

Finally, we excluded individuals on lipid-lowering medication, a potential population that could benefit from earlier screening and intervention using the caIRS. Long-term, prospective studies are needed to determine if identifying individuals at higher genetic risk at an earlier age and before statin initiation has an impact on CAD outcomes.

In summary, our study adds to the growing evidence that genetics can meaningfully improve the accuracy of CAD risk prediction, beyond traditional clinical risk factors, in individuals of diverse ancestries. The CAD caIRS, a tool that combines clinical and genetic risk, has the potential to improve the identification of individuals at high risk for CAD, particularly in populations underrepresented in current risk assessment tools, such as Black/African-American population. Future research should focus on validating the CAD caIRS in larger diverse populations and assessing its utility in guiding clinical decisions for primary prevention of CAD.

## Methods

### Study populations

We used genotype and phenotype data from multiple cohorts to develop and validate the CAD caIRS. These cohorts included the UK Biobank (UKB), Multi-Ethnic Study of Atherosclerosis (MESA, dbGAP study phs000209.v13.p3), Atherosclerosis Risk in Communities study (ARIC, dbGAP study phs000280.v8.p2), Hispanic Community Health Study (HCHS, dbGAP study phs000810.v1.p1), Cardiovascular Health Study (CHS, dbGAP study phs000287.v7.p1), Jackson Heart Study (JHS, dbGAP studies phs000286.v6.p2), and Penn Medicine BioBank (PMBB)^33^. Written informed consent was obtained from all participants prior to their inclusion in each cohort study. Individuals in ARIC and CHS were part of the training cohort for the PCE model^4^. However, given that our validation focused on comparison of the performance of the caIRS to the PCE (and CHS was not used for validation), we do not expect it to be biased.

The UK Biobank (UKB) is a large, prospective, cohort study of the causes, treatment, and prevention of common complex disease^34,35^. Between 2006 and 2010, the study enrolled over 500,000 individuals aged 40–69 years from the general population of the United Kingdom. At enrollment, participants completed a detailed questionnaire to self-report sex, ancestry, lifestyle factors, and environmental exposures and underwent extensive physical examination, including cardiac imaging and monitoring, and collection and storage of biological samples (blood, urine, and saliva)^36,37^. Participants have now been followed up for over a decade and have a wide range of biomarker and genetic data, including whole exome and genome sequences, available for all 500,000 participants^35^.

The Multi-Ethnic Study of Atherosclerosis (MESA, dbGAP study phs000209.v13.p3) is a medical research study investigating the prevalence, correlates, and progression of subclinical cardiovascular disease (CVD) in a population-based sample of more than 6000 men and women aged 45-84 years and free of CVD at baseline from 6 communities in the United States (New York, NY; Baltimore, MD; Chicago, IL; Los Angeles, CA; Twin Cities, MN; and Winston Salem, NC). Recruitment took place between 2000 and 2002^38,39^. The cohort is approximately 38% White, 28% African-American, 22% Hispanic, and 12% Asian, predominantly of Chinese descent. Extensive cohort data were collected over 6 exams, with participants contacted every 9 to 12 months during the study to assess clinical morbidity and mortality. The study collected a comprehensive set of data for standard coronary risk factors and various aspects of cardiovascular health as well as sociodemographic factors, lifestyle factors, and psychosocial factors. Selected measures of subclinical disease and risk factors were repeated at follow-up visits through 2018 allowing study of the progression of disease. Blood samples, DNA, and lymphocytes were collected and preserved. Participants are followed for identification and characterization of CVD events, including acute myocardial infarction and other coronary heart disease, stroke, peripheral vascular disease, and congestive heart failure; therapeutic interventions for CVD; and mortality^40,41^.

The Atherosclerosis Risk in Communities study (ARIC, dbGAP study phs000280.v8.p2) is a prospective epidemiologic study (1987 to present) conducted in 4 US communities (Forsyth County, NC; Jackson, MS; the northwest suburbs of Minneapolis, MN; and Washington County, MD). The ARIC is investigating the etiology and natural history of atherosclerosis, the etiology of clinical atherosclerotic diseases, and variation in cardiovascular risk factors, medical care, and disease by race, gender, location, and date. The ARIC study includes 2 components: a cohort and community surveillance. The present study used data from the cohort component. In the cohort component, over 15,000 participants, aged 45-64 years, were recruited between 1987–1989 and received an extensive examination, including medical, social, and demographic data. Data collection took place during 7 clinic visits between 1987 and 2019^42^.

The Hispanic Community Health Study / Study of Latinos (HCHS/SOL, dbGAP study phs000810.v1.p1) is a prospective, multi-center, epidemiologic study in Hispanic/Latino populations to determine the prevalence of chronic conditions (eg, CVD, diabetes, and pulmonary disease), identify risk and protective factors, and quantify all-cause mortality, fatal and non-fatal CVD and pulmonary disease, and pulmonary disease exacerbation over time. Between 2008 and 2011, over 16,000 men and women, aged 18–74 years, of Cuban, Dominican, Mexican, Puerto Rican, Central American, and South American backgrounds were recruited through 4 centers (Miami, Florida; Bronx, New York; Chicago, Illinois; and San Diego, California). Participants underwent an extensive baseline clinical examination, including biological, behavioral, and socio-demographic assessments. To determine a range of health outcomes, participants underwent 2 additional clinic visits between 2014 and 2017 and 2020 and 2023 and have annual follow-up interviews^43,44^.

The Cardiovascular Health Study (CHS, dbGAP study phs000287.v7.p1) is a prospective study of risk factors for development and progression of coronary heart disease and stroke in people aged ≥65 years. The study enrolled approximately 6000 participants from 4 US communities (Forsyth County, NC; Sacramento County, CA; Washington County, MD; and Pittsburgh, PA) between 1989 and 1990 and a supplemental cohort of 687 predominantly African-American participants between 1992 and 1993. The study participants have undergone extensive clinic examinations for evaluation of markers of subclinical CVD at study baseline and at annual visits through 1998-1999 and again in 2005–2006. They have also been followed up every 6 months by phone to identify cardiovascular events and to assess physical and cognitive health.

The Jackson Heart Study (JHS, dbGAP studies phs000286.v6.p2) is a large, prospective, community-based, observational study investigating environmental and genetic factors associated with CVD among African Americans. Between 2000 and 2004, over 5000 participants, aged 35–84 years, were recruited from urban and rural areas of 3 counties that comprise the Jackson, MS metropolitan statistical area^45,46^. Participants underwent 3 extensive clinical examinations (Exam 1, 2000–2004; Exam 2, 2005–2008; and Exam 3, 2009–2013) that collected data on traditional and putative cardiovascular disease risk factors and measures of subclinical CVD, including echocardiography, cardiac magnetic resonance imaging, and computed tomography scans, and collection of biological samples (i.e., blood, urine, DNA, lymphocytes). Participants receive annual telephone follow-up and have ongoing surveillance of hospitalizations for cardiovascular events and of deaths^47^.

The Penn Medicine BioBank (PMBB) is an electronic health record (EHR)-linked biobank maintained at the University of Pennsylvania. The PMBB was established in 2013 and includes a large variety of health-related information including diagnosis codes, laboratory measurements, imaging data, and lifestyle information as well as genomic and biomarker data. To date, over 174,000 participants have been enrolled, with approximately 30% of participants being of non-European ancestry. The EHR has a median of 7 years of longitudinal data available on participants^33^.

**Fig. 4:**
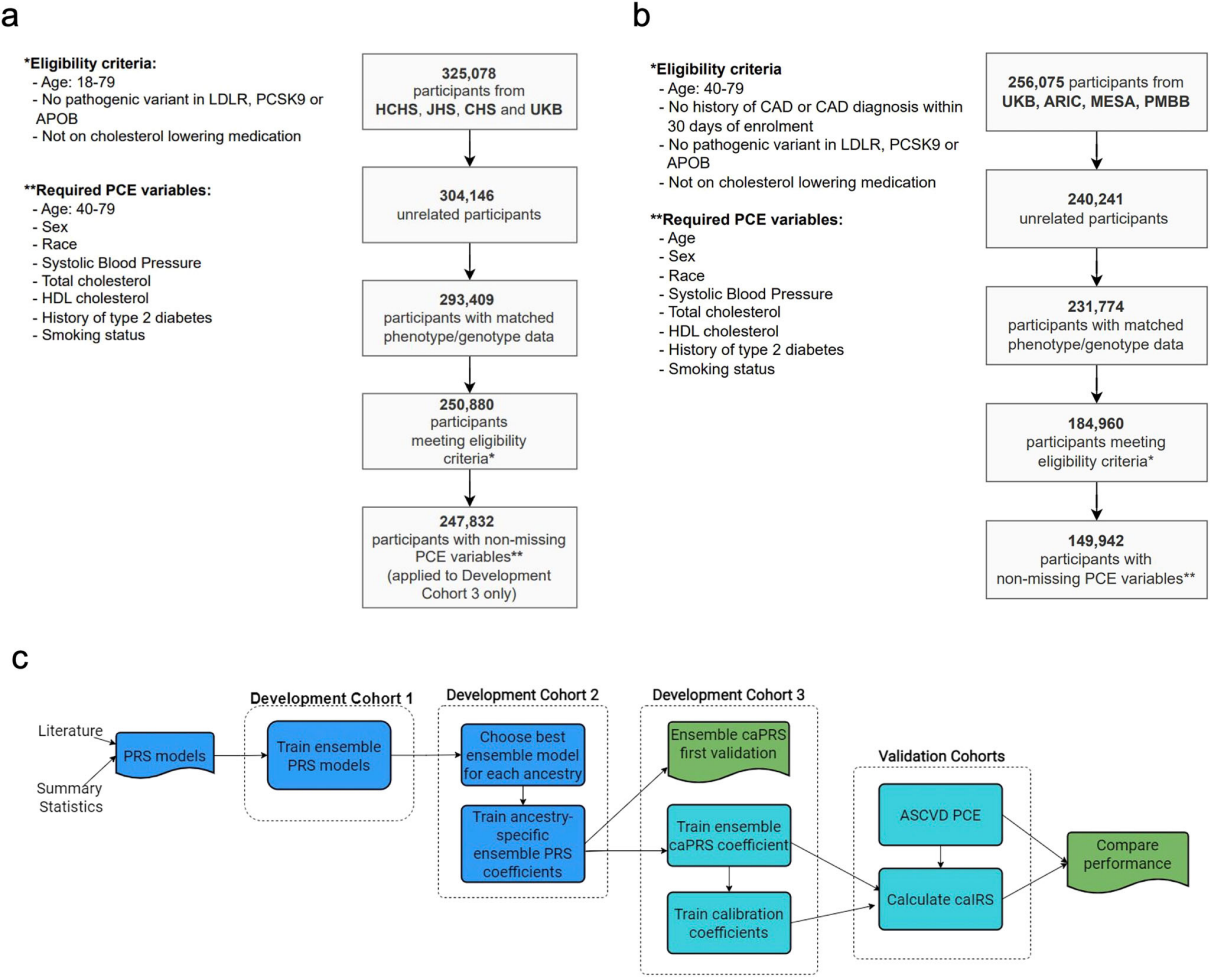
Study cohorts and development/validation workflow. Selection of participants for the caIRS development (**a**) and (**b**) validation. **c** An overview of caIRS development and validation workflow.

We divided the data into 3 development cohorts (Table 3 and Fig. 4) and 4 independent, longitudinal validation cohorts (Table 1 and Fig. 4). The Development Cohorts comprised 26,923 individuals with diagnosed CAD (cases) and 220,909 unaffected individuals (non-cases) from UKB (a subset), HCHS, CHS, and JHS. The Validation Cohorts comprised 11,008 individuals (774 cases) from ARIC, 4162 (240 cases) from MESA, 14 182 (1158 cases) from PMBB, and 120 590 (3050 cases) from UKB who were not included in model development.

### Eligibility criteria

Across all cohorts, we excluded individuals with missing genotype data. In cases where related individuals were present, we retained one individual per pair of first and second-degree relatives. In the UKB cohort, where whole exome sequencing data was available, we additionally excluded individuals with pathogenic or likely pathogenic variants in 3 genes known to cause Familial Hypercholesterolemia (*APOB*, *LDLR*, *PCSK9*). We filtered whole-exome sequencing VCF files to three genes of interest, removed variants in low complexity regions and segmental duplications, and annotated remaining variants using SnpEff, Clinvar, and gnomad. Pathogenic variants were designated in each of the annotations as follows: (1) SnpEff: variants with “HIGH” predicted function effect impact, (2) Clinvar: variants with “Pathogenic” or “Likely pathogenic” clinical significance, (3) Gnomad: variants with <0.5% MAF. Individuals were identified as having a pathogenic variant if they had one or more variants that match these filters.

Individuals in Development Cohorts 1 and 2 were eligible for inclusion if they were between 18 and 79 years of age at the time of diagnosis or assessment for cases and those unaffected, respectively. We also excluded unaffected individuals taking cholesterol-lowering medications. In the case of Development Cohort 3 we additionally restricted the eligible age range to 40–79 and removed individuals missing any of the factors required to calculate the PCE score (Fig. 4).

Individuals in the Validation Cohorts were eligible for inclusion if they had no personal history of CAD and were 40–79 years of age at the time of first assessment. We excluded all individuals taking cholesterol-reducing medication at the time of first assessment, individuals who developed CAD within 30 days of assessment, and affected individuals missing age at diagnosis. Because the CAD caIRS was derived from the PCE, we also excluded individuals missing any data for risk factors included in the PCE model (see Supplementary Table 3). In the case of PMBB where the PCE variables were incomplete (mainly lipid values) for a relatively large subset of eligible individuals (7796 out of 14,182), we imputed missing values using the cohort median to avoid loss of a substantial fraction of the cohort from the analysis. Repeating the PMBB analysis upon exclusion of participants with missing PCE variables (instead of imputing) yielded highly consistent results which we included for comparison (see Supplementary Table 4 and Supplementary Fig. 5).

### Genotype imputation

We combined multiple genotype data sources, which required distinct preprocessing steps depending on the genome build and availability of externally imputed WGS data (see Supplementary Table 5 for dataset-specific preprocessing/imputation steps). In short, we used the existing imputed array genotyping data for UKB and dbGAP datasets, if they were available, and used CrossMap (v0.6.1)^48^ to lift over their coordinates to Genome Reference Consortium Human Build 37 (GRCh37) where necessary. For internal imputation of the array data we converted coordinates to hg19 using array annotation (where necessary), then phased genotypes using SHAPEIT4^49^ and imputed unobserved genotypes with IMPUTE5^50^ using the UK10K reference panel^51^. For WGS datasets we used CrossMap (v0.6.1)^48^ to liftover coordinates to GRCh37. In the case of PMBB, we used the imputed array data (GRCh38) for which genotyping and imputation details have been described previously^33^. In short, for PMBB, we performed genotype imputation using Eagle^52^ and Minimac4^53^ on the TOPMed Imputation Server^54^. For sites that could not be successfully imputed, we obtained a population-specific allele frequency from gnomAD v3.1.1^55^ to estimate the average contribution of the variant when scoring PRSs. For Validation Cohorts, we excluded individuals missing genotypes for more than 5% of PRS sites. In cases where the same participant data was present in multiple datasets within a cohort, we used scores from the dataset with the smallest number of missing PRS sites.

### Use of population descriptors and inference of genetic ancestry

We partitioned individuals from the Development Cohorts (see Model Development below) among 5 ancestry groups based on genetic similarity to one of five continental reference populations: African (AFR), Hispanic/Admixed American (AMR), East Asian (EAS), European (EUR), and South Asian (SAS). Genetic ancestry was decomposed into these five ancestry groups using AIPS^56^ with the 1KGP as a reference panel. We used the 80% ancestral fraction as a threshold to classify participants into “pure” ancestry groups for the purpose of estimating ancestry specific model coefficients (see Model Development).

In order to assess the performance of the caPRS and caIRS in real-world populations, which represent broader genetic diversity than 5 reference population groups, we partitioned validation cohorts based on self-identified ethnicity, without restricting the analysis to genetically “pure” individuals. The following rules were applied to group and standardize self-identified ethnicity labels:

1. Individuals who self-identified as Black, Black British, Caribbean, African, African-American, or any other Black background were labeled as Black/African American (or Black/Black British in case of UKB).
2. Individuals who self-identified as White, Caucasian, White British, Irish, or other White background were labeled as White/Caucasian.
3. Individuals who self-reported as Indian, Pakistani, Bangladeshi were grouped as South Asian (UKB only).
4. Individuals who self-identified as Chinese, Chinese American, or Asian (U.S. cohorts; MESA, PMBB) were labeled as East Asian / Asian American.
5. Individuals who self-identified as Hispanic or Latino were labeled as Hispanic.
6. Finally, individuals with missing ethnicity information or self-identified as other or one of mixed categories were labeled as “Other”.

### Phenotype definitions

CAD designation was standardized across cohorts with diverse phenotypic data as individuals with myocardial infarction (MI), coronary revascularization, or fatal coronary heart disease (CHD). Standardized codes from medical records, such as CAD-related International Classification of Diseases, Tenth Revision (ICD-10), or operation codes, were used when available (I21, I22, I23, I24.1, I25.2). Otherwise, doctor or self-reported data adjudicated by individual cohort committees were used.

### Model development

We undertook a multi-step process to first develop a caPRS for CAD, and then incorporate this caPRS into a clinical prediction model (PCE) that also includes traditional cardiovascular risk factors (Fig. 4). Model development comprised multiple stages including training of candidate PRS models from GWAS summary statistics, training and selecting ancestry-specific PRS ensemble models, estimating ancestry-specific PRS ensemble coefficients for caPRS, and estimating caIRS parameters which include the overall caPRS effect size and calibration coefficients. The steps were followed by the validation of the caPRS and caIRS models performance in independent longitudinal cohorts.

We constructed multiple internal PRS models using multi-ancestry genome-wide association study (GWAS) summary statistics for CAD and type 2 diabetes (T2D), which is a frequently coexisting condition and major risk factor for CAD. We used GWAS summary statistics from CARDIoGRAM (EUR cohort)^16^, Biobank Japan (GWAS cat: GCST90018706; 2 Japanese/EAS cohorts)^17^, Million Veterans Program (dbGAP study phs001672.v9.p1; EUR, AFR, AMR cohorts)^18^, DIAGRAM (GWAS cat: GCST004773; EUR)^19^, GERA (GWAS cat: GCST90086068)^20^, and AFR population metaanalysis (GWAS cat: GCST008114)^21^. PRS models were independently constructed for CAD and T2D using PRS-CSx^57^ with a range of values for the global shrinkage parameter (see Supplementary Table 6 for the list GWAS studies and corresponding parameter values used in development).

PRS centering and standardization were performed as previously described^12^. Briefly, to account for ancestry-specific mean and variance, principal components (PCs) were computed for all individuals by projecting their genotypes onto PCs calculated from the 1KGP dataset using the R package bigsnp^58^. Each ancestry-specific PRS was then centered by subtracting the PRS predicted from a linear regression of PRS against the first four PCs in unaffected individuals^59^. The centered PRS was subsequently divided by the standard deviation (SD) of the corresponding 1KGP population. The scores of PGS catalog models, derived from GWASs independent of the validation cohorts, for CAD and T2D models were linearly combined with PRS-CSx models developed internally. The optimal mixing weights for PRSs were independently learned for each (genetically inferred) continental ancestry group (AFR, AMR, EAS, EUR, SAS) using Development Cohort 1 (Table 3) via the Elastic Net using the LogisticRegressionCV function from the sklearn (Python) package with the following hyperparameters: penalty=“elasticnet”, solver=“saga”, L1_ratios = [0.0, 0.2, 0.4, 0.6, 0.8, 1], scoring=“roc_auc”. In case of ancestral groups originating from multiple cohorts (AFR: UKB + JHS, AMR: UKB + HCHS) an additional term was included in the elastic net model to adjust for the cohort effect. See the Supplementary Table 6 for the list of internally and externally developed PRS models whose scores were used for ensemble development and their corresponding weights.

The performance of the ancestry-specific ensemble models was compared to the ensemble derived from EUR individuals (largest sample) in the Development Cohort 2 (Table 3) across five ancestry groups, using multivariable logistic regression adjusted for age at enrollment, sex, and first-degree family history of CAD/CVD (Supplementary Fig. 6).

### Cross ancestry polygenic risk score (caPRS)

We used the best-performing ensemble model for each ancestry to construct the caPRS. The caPRS is defined as a linear combination of the (ensemble) PRS, multiplied by the fractional ancestry estimate and the PRS effect size, which was estimated for each continental ancestry group from the Development Cohort 2 using multivariable logistic regression, adjusting for age at enrollment, sex, first-degree family history of CAD (where available) and cohort. This methodology follows the previously described caPRS model for Breast Cancer risk prediction^12^ and makes use of continuous genetic ancestry estimates, when calculating the PRS score, obfuscating the need for fixed ancestry labels at prediction time and naturally accommodating genetically admixed individuals. We modified our original method by replacing single best-performing PRS for each population with the best-performing ensemble PRS score. More specifically the caPRS is defined as:

1 $${caPRS}=\mathop{\sum}\limits_{i=1}^{5}{\beta }_{i}* {f}_{i}* {{PRS\; ensemble}}_{i},$$

where ${\beta }_{i}$, ${f}_{i}$ and ${{PRS\; ensemble}}_{i}$ correspond to PRS effect size, fractional ancestry estimate, and selected PRS ensemble score, respectively, for each continental ancestry group *i*.

### Cross ancestry integrated risk score (caIRS)

We estimated the effect size associated with the caPRS in Development Cohort 3 using a multivariable logistic regression including caPRS, age at enrollment, sex, and cohort. We then calculated a calibration constant which depends on the absolute 10-year risk estimate from the PCE model. Finally, we calculated the 10-year CAD risk based on the PCE and caIRS (PCE combined with the caPRS).

The caIRS combines genetic and clinical information and is defined as follows:

2 $${caIRS}=1-{\left(1-{PCE}\right)}^{\exp \left(\beta\, *\, {caPRS}+{C}_{k}\right)}$$

where PCE is the 10-year risk calculated using the PCE algorithm, *β* is the effect size associated with caPRS estimated using Development Cohort 3 via a logistic regression model adjusted for age at enrollment, sex, and cohort and C_k_ corresponds to a calibration constant which depends on the absolute 10-year risk estimate from the PCE model. More specifically, each C_k_ was calculated using unaffected individuals from the Development Cohort 3 within the strata formed by the deciles of the PCE score, such that the average risk of CAD predicted by the caIRS aligns with the average risk predicted by the PCE alone for unaffected controls within each strata. This means that the average contribution of the PRS to the integrated score within a decile is 1, namely:

3 $$E\,[\exp ({beta}* {caPRS})+{C}_{k}]=1$$

for each group k.

The distributions of caPRS, PCE, and caIRS scores are provided in the Supplementary Content (Supplementary Fig. 7).

### Model validation

We evaluated the performance of the caPRS and caIRS using validation cohorts that were independent from those used to generate caPRS and caIRS models. According to expert recommendations regarding reporting of polygenic scores in risk prediction studies^60^, we evaluated our models by considering measures of model discrimination, calibration, and effect size. Associations of the caPRS with 10-year CAD risk were evaluated in terms of HR per SD increase in the caPRS with a 95% confidence interval from multivariable Cox proportional-hazards (PH) models adjusted for age at enrollment and sex. The test statistic was the change in the likelihood deviance metric between the full model and the appropriate reduced model. The C-index was used to assess model discrimination. In situations where specific ancestries lacked sufficient statistical power for reliable analyses, we opted to integrate the effect sizes across studies using meta-analyses, suitably adjusting for the heterogeneity observed among the studies being combined. This approach enabled us to enhance the robustness and generalizability of our findings by leveraging the collective power of multiple datasets. We used a Mantel-Cox log rank test, with caIRS coded as a binary variable, to evaluate whether 10-year CAD incidence was significantly lower for patients at borderline/intermediate PCE risk (5-20%) with a caIRS score below vs above the pre-specified risk threshold (20%). Proportional hazards assumptions were verified for all proposed models using tests and graphs based on the Schoenfeld residuals. We used the Kaplan-Meier method to estimate the cumulative incidence rates of 10-year CAD for subjects above/below the caIRS score threshold. For caIRS and PCE, we assessed net reclassification improvement (NRI), sensitivity, specificity, positive predictive value (PPV), and negative predictive value (NPV) using a 20% high risk threshold^4^. For additional comparison we also evaluated performance using alternative risk classification thresholds of 7.5% and 10%. We assessed PCE and caIRS model calibration by inspecting the concordance between the observed and predicted risks visually and quantitatively via the estimation of calibration intercepts and slopes. To estimate these parameters, we performed logistic regression with the predicted probabilities $(\underline{Y})$ as the independent variable and the observed outcomes $(Y)$ as the dependent variable. More specifically, the calibration intercept $({{\rm{\beta }}}_{0})$ was estimated using the following model: $logit(Y)\,={\beta }_{0}\,+\,logit(\underline{Y})$ and the calibration slope $({{\rm{\beta }}}_{1})$ from: $logit(Y)\,={\beta }_{0}\,+{\beta }_{1}\,*\,logit(\underline{Y})$.

All analyses were performed using R Statistical Software (v4.1.0 or higher)^61^.

## Supplementary information

Supplementary Material

## Data Availability

Datasets used for the analyses described in this manuscript were obtained from UK Biobank Resource under Application Number 48991 and dbGaP at http://www.ncbi.nlm.nih.gov/sites/entrez?db=gap through dbGaP accession study numbers phs000209.v13.p3, phs000280.v8.p2, phs000810.v1.p1, phs000287.v7.p1, and phs000286.v6.p2. The Penn Medicine Biobank genetic data was generated by Regeneron Genetics and made available to study authors for model validation by Penn Medicine Biobank with the permission of Regeneron Genetics.
