# Supplementary material for: Polygenic risk scores improve CAD risk prediction in individuals at borderline and intermediate clinical risk
Source: NPJ Cardiovasc Health. 2025 May 1;2:13. doi: 10.1038/s44325-025-00049-7 (PMC12912298; doi:10.1038/s44325-025-00049-7)
Supplement: Supplementary file 1 — Supplementary Material [file 44325_2025_49_MOESM1_ESM.pdf]

Supplementary Material

Contents:

Supplementary Tables:

- **Supplementary Table 1**: Classification metric values (for caIRS vs PCE) at additional high risk classification thresholds.
- **Supplementary Table 2**: Classification metrics of the ASCVD-PCE and caIRS models using 20% threshold across self-declared ethnicity groups.
- **Supplementary Table 3**: Characteristics of individuals excluded from validation cohorts due to missing PCE variables. See the corresponding Table 1 in the main text which shows the characteristics after excluding participants with missing PCE variables in ARIC, MESA and UKB cohorts.
- **Supplementary Table 4**: Classification metrics of the PCE and caIRS models using 20% threshold in the PMBB cohort after excluding individuals missing PCE variables (instead of imputing).
- **Supplementary Table 5**: An overview of preprocessing and imputation steps for each genomic dataset used in the study.
- **Supplementary Table 6**: List of GWASs used for internal model development with the corresponding PRS-CSx training parameters and final ensemble weights of intermediate models after combining with externally trained PGS catalog models.

Supplementary Figures:

- **Supplementary Figure 1:** PRS stratified cumulative incidence of CAD among the low (PCE<5%) risk group
- **Supplementary Figure 2:** PRS stratified cumulative incidence of CAD among the high (PCE≥20%) risk group.
- **Supplementary Figure 3:** Performance comparison for the caPRS and selected PGS catalog models.
- **Supplementary Figure 4:** Calibration plots for the baseline PCE and caIRS models.
- **Supplementary Figure 5**: 10-year cumulative incidence of CAD among individuals identified as borderline or intermediate risk using PCE and those reclassified into high and low risk groups by caIRS within the PMBB cohort after excluding individuals with missing PCE variables (instead of imputing).
- **Supplementary Figure 6:** Comparative performance of PRS ensemble scores developed in European and ancestry-matched participants.
- **Supplementary Figure 7:** Distributions of the caPRS, PCE and caIRS scores across validation cohorts.
- **Supplementary Figure 8:** Overlay of genetically inferred ancestry labels on genetic principal components in validation cohorts.

Strobe checklist for cohort studies

Supplementary Tables

Supplementary Table 1. Classification metric values (for caIRS vs PCE) at additional high risk classification thresholds.

Threshold (%)	Cohort	N (cases)	Model	NRI (%)	Sensitivity (%)	Specificity (%)	PPV (%)	NPV (%)
7.5	ARIC	11008 (774)	PCE	-	80.5	57.2	12.4	97.5
			caIRS	4.42 (1.74 - 6.98)	79.6	62.5	13.8	97.6
	MESA	4162 (240)	PCE	-	82.1	49.8	9.1	97.8
			caIRS	0.86 (-3.71 - 5.19)	76.7	56.0	9.6	97.5
	PMBB	14182 (1158)	PCE	-	72.7	62.6	14.8	96.3
			caIRS	3.68 (1.46 - 5.67)	73.1	65.9	16.0	96.5
	UKB	120590 (3050)	PCE	-	75.2	64.5	5.2	99.0
			caIRS	5.83 (4.53 - 7.3)	77.6	68.0	5.9	99.2
10	ARIC	11008 (774)	PCE	-	68.1	69.1	14.3	96.6
			caIRS	5.76 (3.01 - 8.70)	70.5	72.4	16.2	97.0
	MESA	4162 (240)	PCE	-	74.6	58.9	10.0	97.4
			caIRS	-1.47 (-5.94 - 2.97)	67.5	64.5	10.4	97.0
	PMBB	14182 (1158)	PCE	-	61.7	72.0	16.4	95.5
			caIRS	6.00 (3.85 - 8.17)	65.3	74.5	18.5	96.0
	UKB	120590 (3050)	PCE	-	63.2	74.4	6.0	98.7
			caIRS	5.76 (4.33-7.29)	67.2	76.2	6.8	98.9

Supplementary Table 2. Classification metrics of the ASCVD-PCE and caIRS models using 20% threshold across self-reported ethnicity groups.

Model	Ethnicity	Cohort	N (cases)	NRI	Sensitivity	Specificity	PPV	NPV	C-index
PCE	Hispanic	MESA	828 (48)	-	43.8	80	11.9	95.8	0.765 (0.715-0.816)
caIRS				12.08 (4.12-21.75)	54.2	81.7	15.4	96.7	0.783 (0.731-0.834)
PCE		PMBB	803 (78)		26.9	92.7	28.4	92.2	0.739 (0.691-0.787)
caIRS				16.16 (6.12-25.81)	44.9	90.9	34.6	93.9	0.79 (0.744-0.835)
PCE	Black/African American	ARIC	2559 (167)	-	45.5	85.9	18.4	95.8	0.757 (0.722-0.793)
caIRS				3.86 (-0.52-8.33)	49.7	85.5	19.4	96.1	0.762 (0.726-0.797)
PCE		MESA	1067 (61)	-	59	80.7	15.7	97	0.792 (0.745-0.839)
caIRS				1.09 (-5.39-7.56)	59	81.8	16.4	97	0.785 (0.735-0.835)
PCE		PMBB	3078 (173)	-	23.4	92.7	15.8	95.4	0.72 (0.682-0.758)
caIRS				1.68 (-3.05-6.68)	25.3	92.5	16.5	95.5	0.732 (0.694-0.77)
PCE		UKB	1842 (23)	-	30.4	92.2	4.7	99	0.755 (0.672-0.839)
caIRS				0.38 (-0.22-0.94)	30.4	92.6	4.9	99.1	0.764 (0.685-0.843)
PCE	East Asian / Asian American	MESA	526 (15)	-	33.3	80	4.7	97.6	0.702 (0.619-0.784)
caIRS				9.8 (-13.34-34.06)	40	83.2	6.5	97.9	0.752 (0.655-0.849)
PCE		PMBB	221 (17)	-	23.5	95.1	28.6	93.7	0.659 (0.543-0.774)
caIRS				-7.84 (-21.17–0.48)	17.6	93.1	17.6	93.1	0.698 (0.6-0.797)
PCE	White/ Caucasian	ARIC	8449 (607)	-	24	93.9	23.3	94.1	0.766 (0.75-0.782)
caIRS				11.03 (7.66-14.58)	36.9	92	26.4	95	0.795 (0.779-0.811)
PCE		MESA	1741 (116)	-	28.4	83.8	11.1	94.2	0.675 (0.631-0.719)
caIRS				5.35 (-3.41-14.21)	30.2	87.4	14.6	94.6	0.7 (0.657-0.742)
PCE		PMBB	10615 (928)	-	30.7	90.7	24.2	93.2	0.737 (0.722-0.752)
caIRS				10.04 (7.07-12.82)	41.2	90.3	29	94.1	0.765 (0.75-0.78)
PCE		UKB	113904 (2895)	-	25.7	93.7	9.7	98	0.767 (0.76-0.775)
caIRS				9.92 (8.36-11.47)	38	91.4	10.3	98.3	0.798 (0.79-0.805)
PCE	(South) Asian or Asian British	UKB	1766 (69)	-	10.1	93.5	5.9	96.2	0.734 (0.685-0.782)
caIRS				15.03 (6.11-24.24)	27.5	91.1	11.2	96.9	0.779 (0.734-0.824)
PCE	Other	UKB	2669 (57)	-	31.6	95.6	13.6	98.5	0.785 (0.731-0.838)
caIRS				4.69 (-5.4-15.03)	36.8	95.1	14	98.6	0.805 (0.755-0.856)

Supplementary Table 3. Characteristics of individuals excluded from validation cohorts due to missing PCE variables. See the corresponding Table 2 in the main text which shows the characteristics after excluding participants with missing PCE variables in ARIC, MESA and UKB cohorts.

ARIC												
**Self-reported ethnicity**	**Total Count**	**Men**		**Women**		**Age***	**Follow up***	**Total Cholesterol***	**HDL Cholesterol***	**Systolic Blood Pressure***	**Diabetes***	**Current Smoker***
		**Cases**	**Noncases**	**Cases**	**Noncases**							
Black/African American	133	2 (5.4%)	35 (94.6%)	4 (4.2%)	92 (95.8%)	52.0 (10.0)	24.0 (14.0)	210.0 (34.8)	35.3 (14.4)	126.0 (29.5)	22 (16.5%)	32 (24.1%)
White/Caucasian	32	2 (12.5%)	14 (87.5%)	0 (0%)	16 (100%)	51.5 (11.0)	23.5 (13.2)	201.0 (69.8)	32.7 (6.4)	121.0 (22.5)	2 (6.2%)	5 (15.6%)
All	165	4 (7.5%)	49 (92.5%)	4 (3.6%)	108 (96.4%)	52.0 (10.0)	24.0 (14.0)	206.0 (62.0)	32.7 (8.5)	124.5 (28.2)	24 (14.5%)	37 (22.4%)
**MESA**												
**Self-reported ethnicity**	**Total Count**	**Men**		**Women**		**Age***	**Follow up***	**Total Cholesterol***	**HDL Cholesterol***	**Systolic Blood Pressure***	**Diabetes***	**Current Smoker***
		**Cases**	**Noncases**	**Cases**	**Noncases**							
Black/African American	14	0 (0%)	5 (100%)	0 (0%)	9 (100%)	60.0 (14.0)	9.8 (4.2)	178.0 (63.0)	53.5 (21.0)	130.2 (25.6)	1 (7.1%)	0 (0.0%)
Hispanic	1	0 (0%)	0 (0%)	0 (0%)	1 (100%)	58.0 (0.0)	10.7 (0.0)	NA (NA)	NA (NA)	108.5 (0.0)	0 (0.0%)	0 (0.0%)
East Asian/Asian American	1	0 (0%)	1 (100%)	0 (0%)	0 (0%)	51.0 (0.0)	10.7 (0.0)	NA (NA)	NA (NA)	116.0 (0.0)	0 (0.0%)	0 (0.0%)
White/Caucasian	13	0 (0%)	6 (100%)	0 (0%)	7 (100%)	65.0 (13.0)	9.9 (0.5)	189.0 (49.0)	50.0 (11.0)	122.0 (16.5)	1 (7.7%)	1 (7.7%)
All	29	0 (0%)	12 (100%)	0 (0%)	17 (100%)	60.0 (14.0)	10.0 (0.6)	183.5 (52.3)	50.0 (13.0)	122.0 (24.2)	2 (6.9%)	1 (3.4%)
**UKB**												
**Self-reported ethnicity**	**Total Count**	**Men**		**Women**		**Age***	**Follow up***	**Total Cholesterol***	**HDL Cholesterol***	**Systolic Blood Pressure***	**Diabetes***	**Current Smoker***
		**Cases**	**Noncases**	**Cases**	**Noncases**							
Black/Black British	317	1 (0.8%)	130 (99.2%)	2 (1.1%)	184 (98.9%)	49.0 (9.0)	12.1 (1.3)	200.0 (48.1)	54.4 (19.5)	133.0 (24.8)	43 (13.6%)	35 (11.0%)
East Asian/Asian American	86	1 (3.2%)	30 (96.8%)	0 (0%)	55 (100%)	51.0 (11.0)	12.9 (1.3)	208.6 (45.4)	52.8 (19.7)	128.5 (26.8)	9 (10.5%)	6 (7.0%)
White/Caucasian	25539	455 (4.4%)	9981 (95.6%)	201 (1.3%)	14902 (98.7%)	57.0 (13.0)	12.8 (1.4)	226.0 (54.3)	54.7 (19.0)	134.5 (25.0)	1101 (4.3%)	2662 (10.4%)
Other	750	22 (7%)	291 (93%)	9 (2.1%)	428 (97.9%)	52.0 (12.0)	12.2 (1.5)	222.0 (52.5)	50.7 (20.1)	131.0 (26.0)	57 (10.4%)	69 (12.5%)
(South) Asian or Asian British	336	15 (9.3%)	147 (90.7%)	3 (1.7%)	171 (98.3%)	51.0 (13.0)	12.1 (1.3)	211.7 (52.2)	41.7 (11.3)	132.0 (25.0)	70 (20.8%)	29 (8.6%)
All	27028	494 (4.4%)	10579 (05.6%)	215 (1.3%)	15740 (98.7%)	56.0 (13.0)	12.7 (1.4)	225.3 (54.4)	54.4 (19.1)	134.5 (25.0)	1328 (4.9%)	2801 (10.4%)
*median(IQR); n (%)												

Supplementary Table 4. Classification metrics of the PCE and caIRS models using 20% threshold in the PMBB cohort after excluding individuals missing PCE variables (instead of imputing).

Model	N (cases)	Cohort	NRI	Sensitivity	Specificity	PPV	NPV	C-index
PCE	6386 (608)	PMBB (complete cases)	-	28.12	92.1	27.27	92.4	0.736 (0.717-0.754)
caIRS			7.14 (3.60-10.58)	35.2	92.2	32.1	93.1	0.761 (0.743-0.779)

Supplementary Table 5. An overview of preprocessing and imputation steps for each genomic dataset used in the study.

Dataset	Use existing WGS or array imputed data	Liftover to hg19 using CrossMap	Strand correct hg18 data and convert to hg19 using array annotation	Impute with SHAPEIT4/ IMPUTE5 using UK10K reference	Impute with Eagle/ Minimac4 using TOPMed R2 reference (GRCh38)
ARIC-TOPMed		X			
ARIC-phs000090-II			X	X	
ARIC-phs000090-EA	X				
ARIC-phs000668-WGS	X				
HCHS-phs000880				X	
HCHS-phs001395-TOPMed		X			
JHS-phs000499-CARe			X	X	
JHS-phs000964-TOPMed		X			
CHS-phs001368-TOPMed		X			
MESA-phs000420.v6.p3			X	X	
MESA-phs001416.v2.p1-TOPMed		X			
UKB	X				
PMBB					X*

**Verma A, Damrauer SM, Naseer N, Weaver J, Kripke CM, Guare L et al. The Penn Medicine BioBank: Towards a Genomics-Enabled Learning Healthcare System to Accelerate Precision Medicine in a Diverse Population. J Pers Med 2022; 12. doi:10.3390/jpm12121974.*

Supplementary Table 6. List of GWASs used for internal model development with the corresponding PRS-CSx training parameters and final ensemble weights of intermediate models after combining with externally trained PGS catalog models. GWAS set 1: CARDIoGRAM (GCST000998, EUR) + Sakaue-Kanai (GCST90018657, EAS) + Matsunaga (GCST010480, EAS) + MVP (phs001672.v9.p1, EUR+AFR+AMR), GWAS set 2: CARDIoGRAM (GCST000998, EUR) + Sakaue-Kanai (GCST90018657, EAS) + Matsunaga (GCST010480, EAS), GWAS set 3: CARDIoGRAM (GCST000998, EUR) + MVP (phs001672.v9.p1, EUR), GWAS set 4: MVP (phs001672.v9.p1, AFR+AMR+EAS+EUR) + Sakaue-Kanai (GCST90018657, EAS) + GCST008114 (AFR) + GERA (GCST90086068, EUR).

Input GWAS	PRS-CSx shrinkage param (phi)	Intermediate PRS-CSx model	PGS catalog ID	GWAS phenotype	ensemble weights (optimized in Development Cohort 1)				
					AFR	AMR	EAS	EUR	SAS
GWAS set 1	auto	META	-	CAD	0	-0.1367872005	0.007173950446	0.0458638056	0.01916216289
GWAS set 1	auto	EUR	-	CAD	0	-0.05208162861	0.009543490889	0.00198219982	0.01541532371
GWAS set 1	auto	EAS	-	CAD	0	0.03336093381	0.006160381353	0.02745491748	0.001318475088
GWAS set 1	auto	AMR	-	CAD	0	0.1513095134	0.008400480633	0.02883711029	-0.001949032042
GWAS set 1	auto	AFR	-	CAD	0.03127307136	-0.008173257938	0.00756751547	0.01277339947	-0.00473690512
GWAS set 2	auto	META	-	CAD	0	0.05725312163	0.000565553972	0.01011551415	0.01753701788
GWAS set 2	auto	EUR	-	CAD	0.0159506736	0.01969566545	0.001579099617	0	0.02881577953
GWAS set 2	auto	EAS	-	CAD	0	0.01320156919	0.003585568053	0.03398010559	-0.003819313746
GWAS set 3	1.00E-06	EUR	-	CAD	0	-0.03542451703	0.01132028913	-0.03386888704	0.02598520099
GWAS set 3	1.00E-04	EUR	-	CAD	0	0.02872216956	0.009134458448	0	0.02107121004
GWAS set 3	1.00E-02	EUR	-	CAD	0	0.08086961897	0.003326170764	0	0.01605200077
GWAS set 3	1.00E+00	EUR	-	CAD	0	0.1086751622	0.001122604789	0	0.009962172898
GWAS set 3	1.00E-06	META	-	CAD	0.1112053501	0.1138842234	0.01077256984	-0.0476883235	0.02495277961
GWAS set 3	1.00E-04	META	-	CAD	0	0.03894943351	0.009639974625	0.1340744988	0.02462970861
GWAS set 3	1.00E-02	META	-	CAD	0	0.08304673455	0.003633735931	0.09429582692	0.02238852388
GWAS set 3	1.00E+00	META	-	CAD	0	0.08946218404	0.002369447978	0.05460739932	0.01576854722
GWAS set 3	auto	META	-	CAD	0	-0.04229869769	0.009661357668	0.02821038991	0.02393639977
GWAS set 3	auto	EUR	-	CAD	0	-0.05679953701	0.009222989064	0.02943469942	0.02335789499
GWAS set 4	1.00E-07	META	-	T2D	0	-0.09289088708	-0.0004581258692	0	0.00970319786
GWAS set 4	1.00E-07	EUR	-	T2D	0	0.06261195543	0.003214972004	0	0.008790659187
GWAS set 4	1.00E-07	EAS	-	T2D	0	-0.07185517775	-0.0009207949929	0	0.004735699059
GWAS set 4	1.00E-07	AMR	-	T2D	0	-0.08954770624	-0.002340936331	0	0.009952232157
GWAS set 4	1.00E-07	AFR	-	T2D	0	0.001717636111	0.003908016354	0	0.003566438009
GWAS set 4	1.00E-06	META	-	T2D	0	0.01265974163	-0.003105139856	0	0.007594927953
GWAS set 4	1.00E-06	EUR	-	T2D	0	-0.1354820597	0.002599364814	0	0.005840529569
GWAS set 4	1.00E-06	EAS	-	T2D	0	0.02606254973	-0.002953639815	0	-0.0004836739087
GWAS set 4	1.00E-06	AMR	-	T2D	-0.03699982185	-0.2065575535	-0.001794971954	-0.01358710752	0.008407708535
GWAS set 4	1.00E-06	AFR	-	T2D	0	-0.01403252592	0.002083102552	-0.01141808033	0.004445427604
GWAS set 4	1.00E-05	META	-	T2D	0	0.1621014514	-0.001007801209	0	0.01095545865
GWAS set 4	1.00E-05	EUR	-	T2D	0	0.03134273167	0.003780907449	0	0.001975980217
GWAS set 4	1.00E-05	EAS	-	T2D	0	0.1027221383	-0.001050521295	-0.00975080633	0.004902335126
GWAS set 4	1.00E-05	AMR	-	T2D	0	-0.02116031879	-0.00143073442	0	0.0142524975
GWAS set 4	1.00E-05	AFR	-	T2D	0	0.1316863469	0.004147299132	-0.001385577764	0.01149772548
GWAS set 4	1.00E-04	META	-	T2D	0	-0.01740040614	0.0005115243006	0	0.01152144916
GWAS set 4	1.00E-04	EUR	-	T2D	0	0.07371856667	0.004655109022	0	-0.003482684878
GWAS set 4	1.00E-04	EAS	-	T2D	0	0.07632511875	-0.001416327225	0	0.005084074075
GWAS set 4	1.00E-04	AMR	-	T2D	0	-0.07170297978	-0.0002837284246	0.005150254101	0.008018774352
GWAS set 4	1.00E-04	AFR	-	T2D	0	-0.006094606694	0.0002853488436	0	0.008122554291
GWAS set 4	1.00E-03	META	-	T2D	0	0.0717879142	0.001819185892	0.06522547578	0.01311320347
GWAS set 4	1.00E-03	EUR	-	T2D	0	-0.00019510343	0.004630246372	0.01926778358	-0.006199111932
GWAS set 4	1.00E-03	EAS	-	T2D	0	-0.05603088209	-0.002017760608	0.02704736225	0.00810600878
GWAS set 4	1.00E-03	AMR	-	T2D	0	0.08963333459	0.0002937653276	0	0.00659879294
GWAS set 4	1.00E-03	AFR	-	T2D	0	-0.08510423886	0.0005116416889	0	-0.001357753094
GWAS set 4	1.00E-02	META	-	T2D	0	0.09928902195	0.004911576321	0	0.01391080269
GWAS set 4	1.00E-02	EUR	-	T2D	0	0.01181684326	0.005484475569	0	-0.008925467436
GWAS set 4	1.00E-02	EAS	-	T2D	0.0958710261	-0.1277371482	-0.001610911519	0	0.01056044743
GWAS set 4	1.00E-02	AMR	-	T2D	0	0.102048624	-0.0006493086331	0.01989838979	0.007665366669
GWAS set 4	1.00E-02	AFR	-	T2D	0	0.03220614151	0.001878202519	0	-0.002006978726
-	-	-	PGS002262	CAD	0.05970073118	0.0289280773	0.004753192069	0.01432779066	0.02282254498
-	-	-	PGS000058	CAD	0	0.0608212468	0.008018915109	0.2334567199	0.03674892348
-	-	-	PGS002244	CAD	0	-0.09506296945	0.009541751382	0.04631240141	0.02729346466
-	-	-	PGS001780	CAD	0.02364570333	0.1313180077	0.00639365662	0.1900500093	0.03429458053
-	-	-	PGS000818	CAD	0.07217537681	-0.195374569	0.002268699493	-0.02285928057	0.02746159836
-	-	-	PGS000296	CAD	0	0.2317196629	0.007260699116	0	0.02936631226
-	-	-	PGS002733	T2D	0	0.05286441425	0.0007252994932	0.001481552393	-0.001493367159
-	-	-	PGS002379	T2D	0	0.0541990338	0.01128952988	0.01488201973	0.0002079882239
-	-	-	PGS002243	T2D	0	-0.0606436996	0.004736699095	-0.005989401288	-0.02020286473

Supplementary Figures

Supplementary Figure 1. PRS stratified cumulative incidence of CAD among the low (PCE<5%) risk group. (a) UKB, (b) ARIC, (c) MESA, (d) PMBB.

a

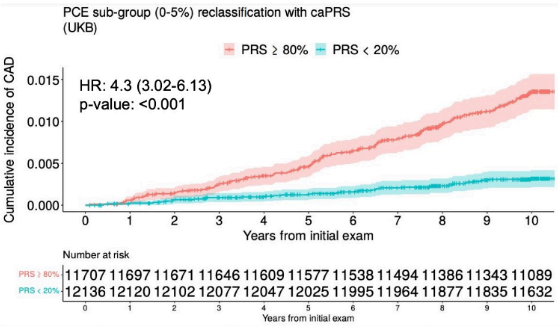

PCE	caPRS	N	N event	Cumulative incidence
0-5%	High PRS (80-100%)	11707	157	1.36 (1.15-1.57)
0-5%	Mid PRS (40-60%)	35606	200	0.57 (0.49-0.65)
0-5%	Low PRS (0-20%)	12136	38	0.32 (0.22-0.42)

b

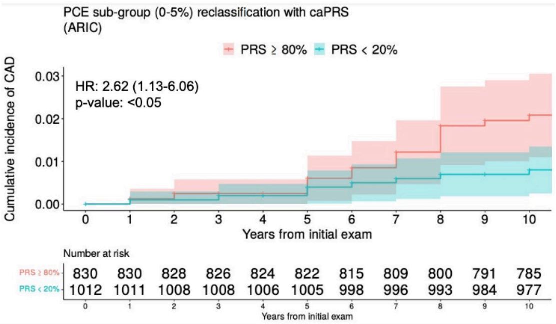

PCE	caPRS	N	N event	Cumulative incidence
0-5%	High PRS (80-100%)	830	17	2.08 (1.1-3.06)
0-5%	Mid PRS (40-60%)	2414	33	1.40 (0.92-1.87)
0-5%	Low PRS (0-20%)	1012	8	0.80 (0.25-1.35)

c

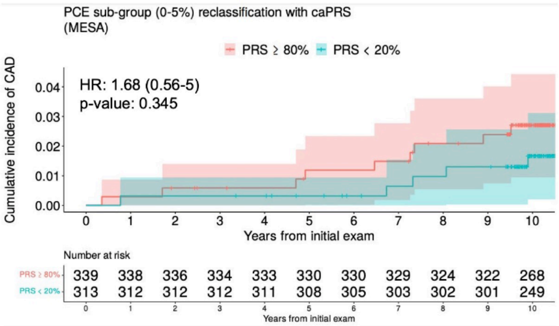

PCE	caPRS	N	N event	Cumulative incidence
0-5%	High PRS (80-100%)	339	9	2.7 (0.95-4.43)
0-5%	Mid PRS (40-60%)	836	11	1.37 (0.56-2.17)
0-5%	Low PRS (0-20%)	313	5	1.67 (0.21-3.12)

d

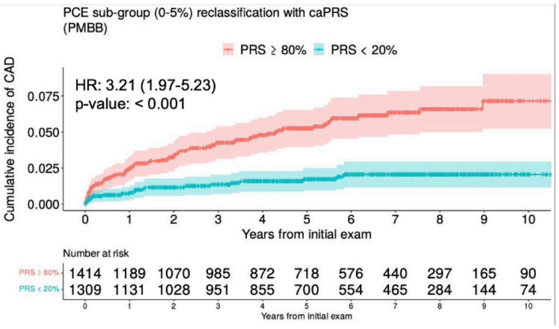

PCE	caPRS	N	N event	Cumulative incidence
0-5%	High PRS (80-100%)	1414	71	7.15 (5.22-9.04)
0-5%	Mid PRS (40-60%)	3920	103	4.21 (3.07-5.33)
0-5%	Low PRS (0-20%)	1309	21	2.05 (1.15-2.95)

Supplementary Figure 2. PRS stratified cumulative incidence of CAD among the high (PCE≥20%) risk group. (a) UKB, (b) ARIC, (c) MESA, (d) PMBB.

a

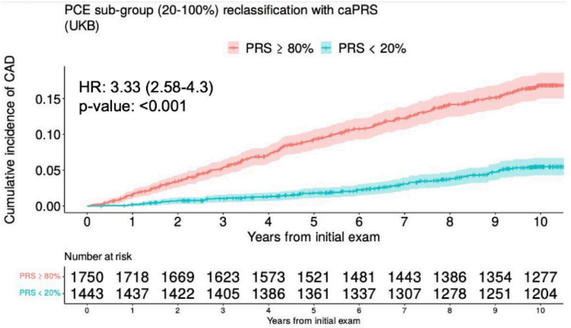

PCE	caPRS	N	N event	Cumulative incidence
20-100%	High PRS (80-100%)	1750	282	16.81 (15-18.59)
20-100%	Mid PRS (40-60%)	4929	421	8.94 (8.12-9.75)
20-100%	Low PRS (0-20%)	1443	75	5.5 (4.28-6.7)

b

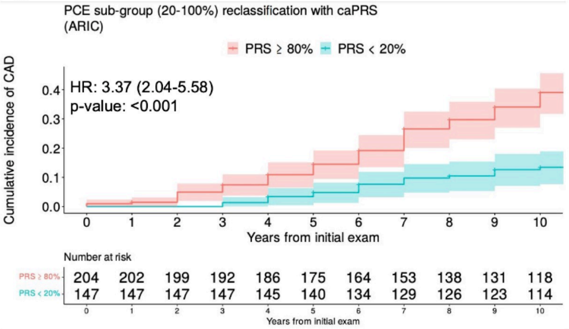

PCE	caPRS	N	N event	Cumulative incidence
20-100%	High PRS (80-100%)	204	75	39.07 (31.71-45.63)
20-100%	Mid PRS (40-60%)	690	128	19.77 (16.63-22.79)
20-100%	Low PRS (0-20%)	147	19	13.44 (7.62-18.89)

c

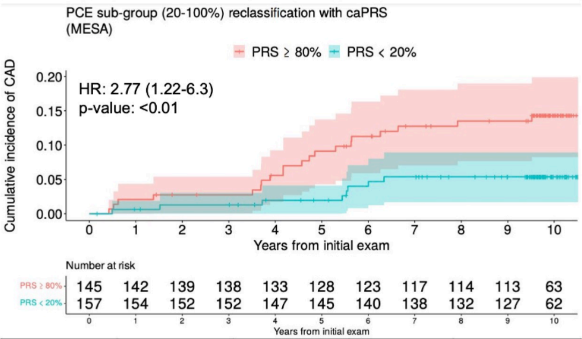

PCE	caPRS	N	N event	Cumulative Incidence
20-100%	High PRS (80-100%)	145	20	14.29 (8.28-19.91)
20-100%	Mid PRS (40-60%)	509	67	14.55 (11.18-17.79)
20-100%	Low PRS (0-20%)	157	8	5.38 (1.68-8.93)

d

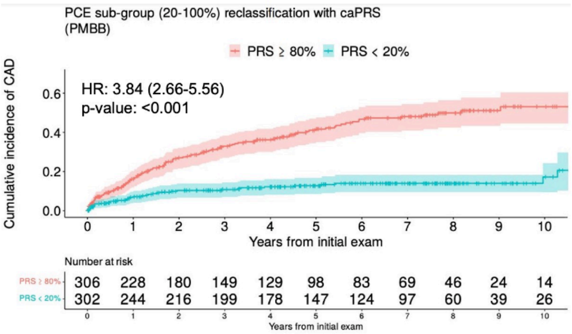

PCE	caPRS	N	N event	Cumulative incidence
20-100%	High PRS (80-100%)	306	120	53.14 (44.51-60.42)
20-100%	Mid PRS (40-60%)	864	182	28.16 (24.19-31.92)
20-100%	Low PRS (0-20%)	302	36	17.15 (9.36-24.27)

Supplementary Figure 3. Performance comparison for the caPRS and selected PGS catalog models, including models used for caPRS (ensemble) development and the recently published GPSMult model (PGS003725_CAD) (Patel et al. 2023). The figure depicts hazard ratios (HRs) for the age and sex-adjusted association between each PRS and 10-year CAD incidence for each self-declared ethnicity group and validation cohort. The top row (META) corresponds to metaanalysis across cohorts. Note that UKB was not included in this analysis because it was part of the development cohort for GPSMult/PGS003725_CAD.

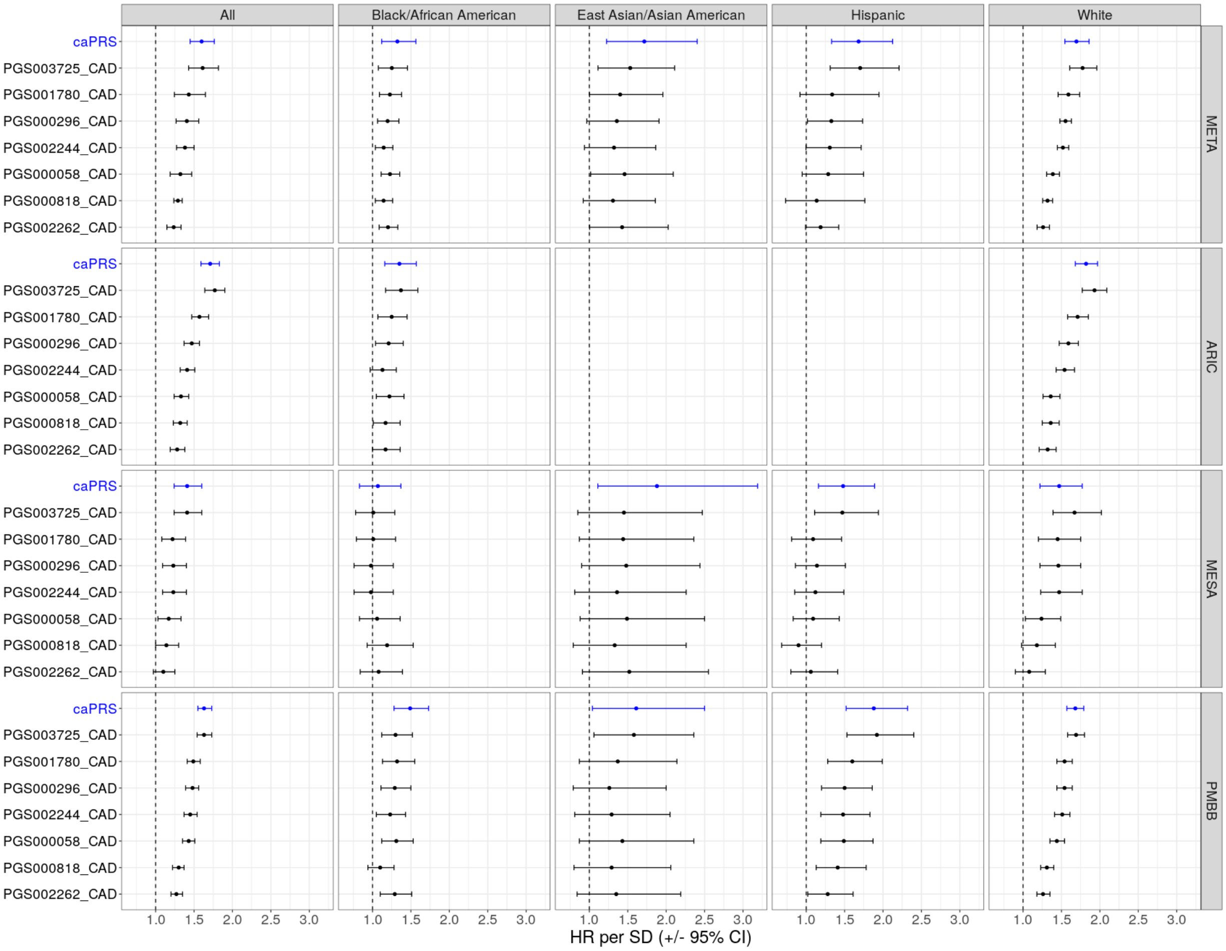

Supplementary Figure 4. Calibration plots for the baseline PCE and caIRS models. The metrics displayed on the plot include calibration intercept (β_0) and slope (β_1) with 95% confidence intervals: (a) UKB, (b) ARIC, (c) MESA, (d) PMBB.

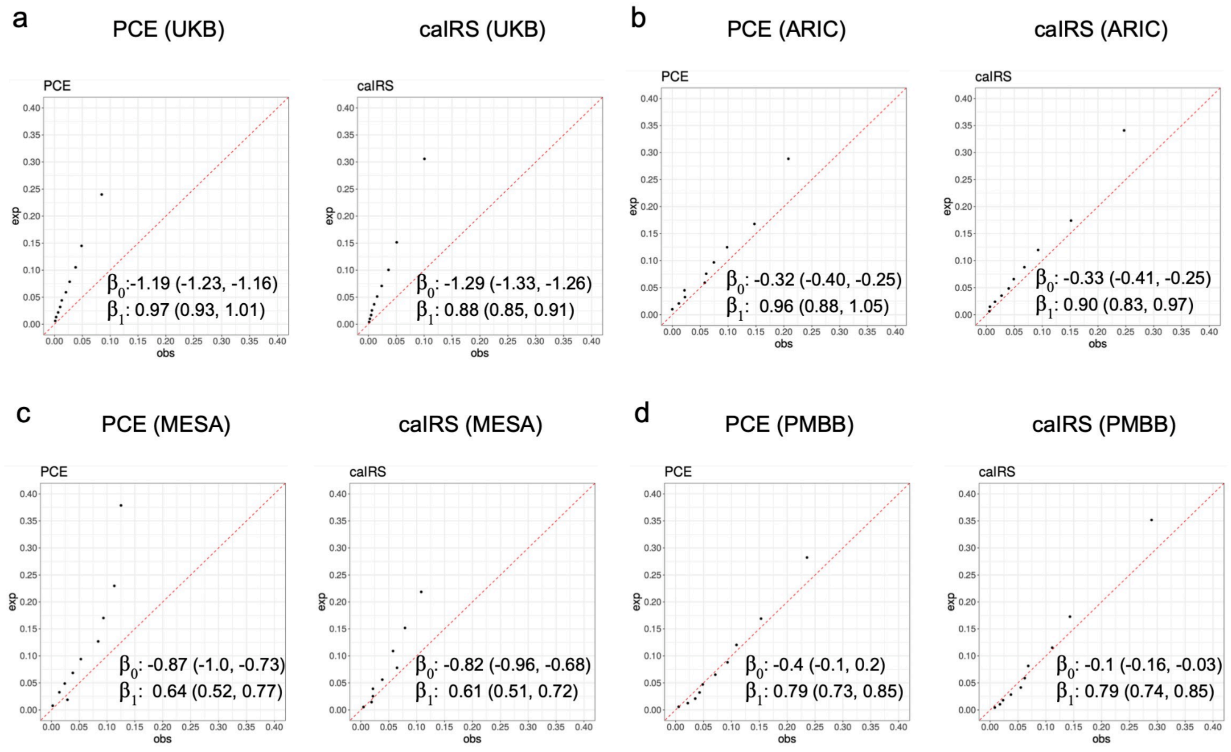

Supplementary Figure 5. 10-year cumulative incidence of CAD among individuals identified as borderline or intermediate risk using PCE and those reclassified into high and low risk groups by caIRS with the corresponding 10-year cumulative incidence rates (+/- 95% CI), counts of individuals (N) and events (N event) for each group. The data corresponds to the PMBB cohort after excluding participants missing PCE variables (instead of imputing). See the corresponding Figure 3D in the main text showing results after imputing missing PCE variables.

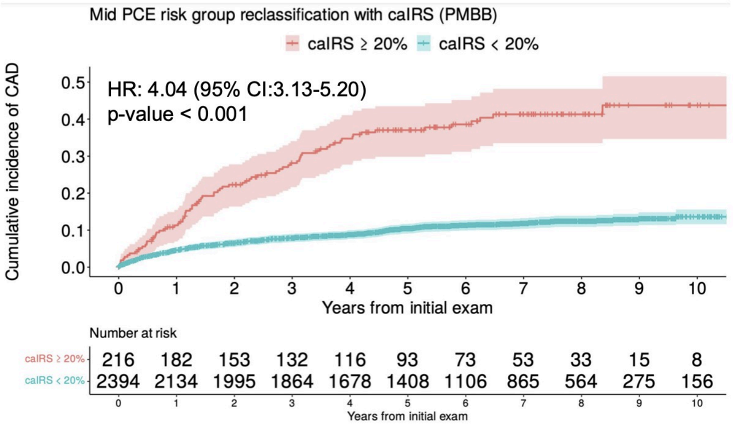

PCE	caIRS	N	N event	Cumulative incidence
5-20%	-	2610 (100%)	329	16.14 (14.15-18.08)
5-20%	<20%	2394 (91.7%)	250	13.62 (11.62-15.56)
5-20%	>=20%	216 (8.3%)	79	43.74 (34.63-51.57)

Supplementary Figure 6. Comparative performance of PRS ensemble scores developed in European (*PRS ensemble (EUR)*) and ancestry-matched participants (*PRS ensemble (ancestry-matched)*) from the Development Cohort 1. Panel headers correspond to genetically inferred ancestry groups: AFR - African, AMR - Admixed American, EAS - East Asian, EUR - European, SAS - South Asian and ALL - all participants from the Development Cohort 2. The figure presents odds ratios per standard deviation, adjusted for age at enrollment, sex, first-degree family history of CAD and cohort (for subgroups originating from multiple cohorts). Note the different x-axis scale for each panel.

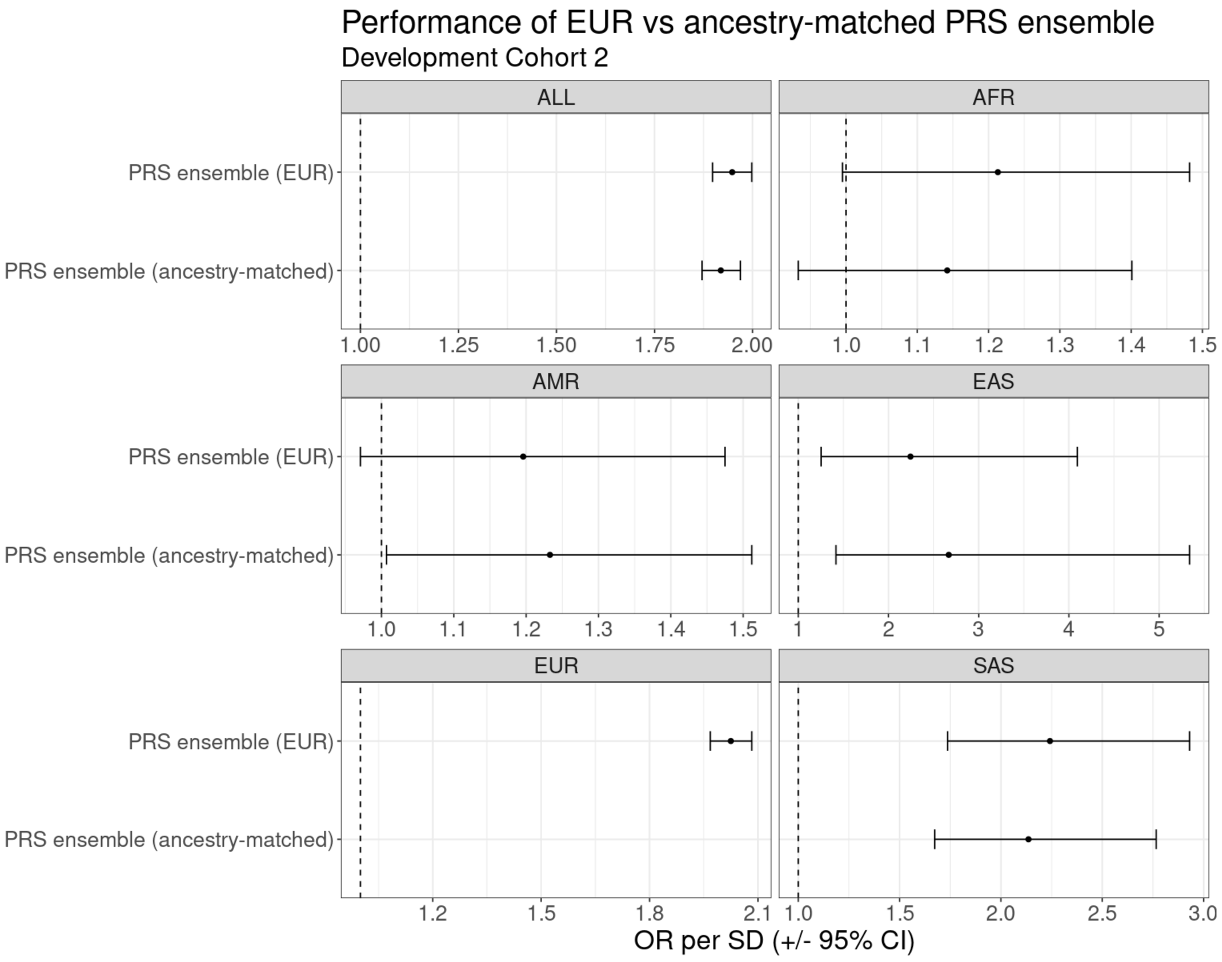

Supplementary Figure 7. Distributions of the caPRS, PCE and caIRS scores across validation cohorts. (a) UKB, (b) ARIC, (c) MESA, (d) PMBB.

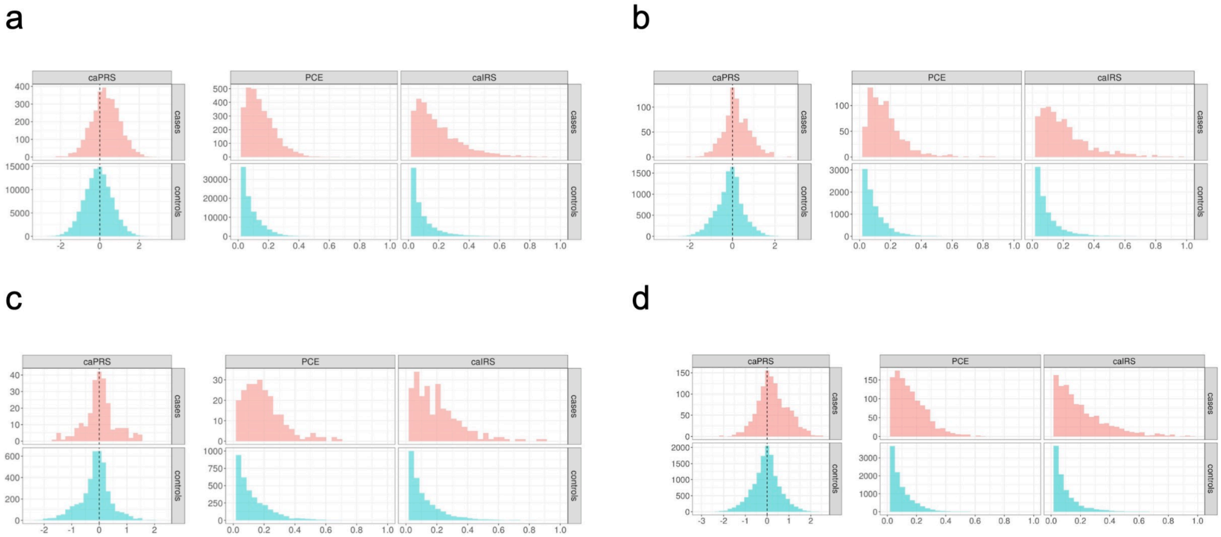

Supplementary Figure 8. Overlay of genetically inferred ancestry labels on genetic principal components in validation cohorts. (a) UKB, (b) ARIC, (c) MESA, (d) PMBB.

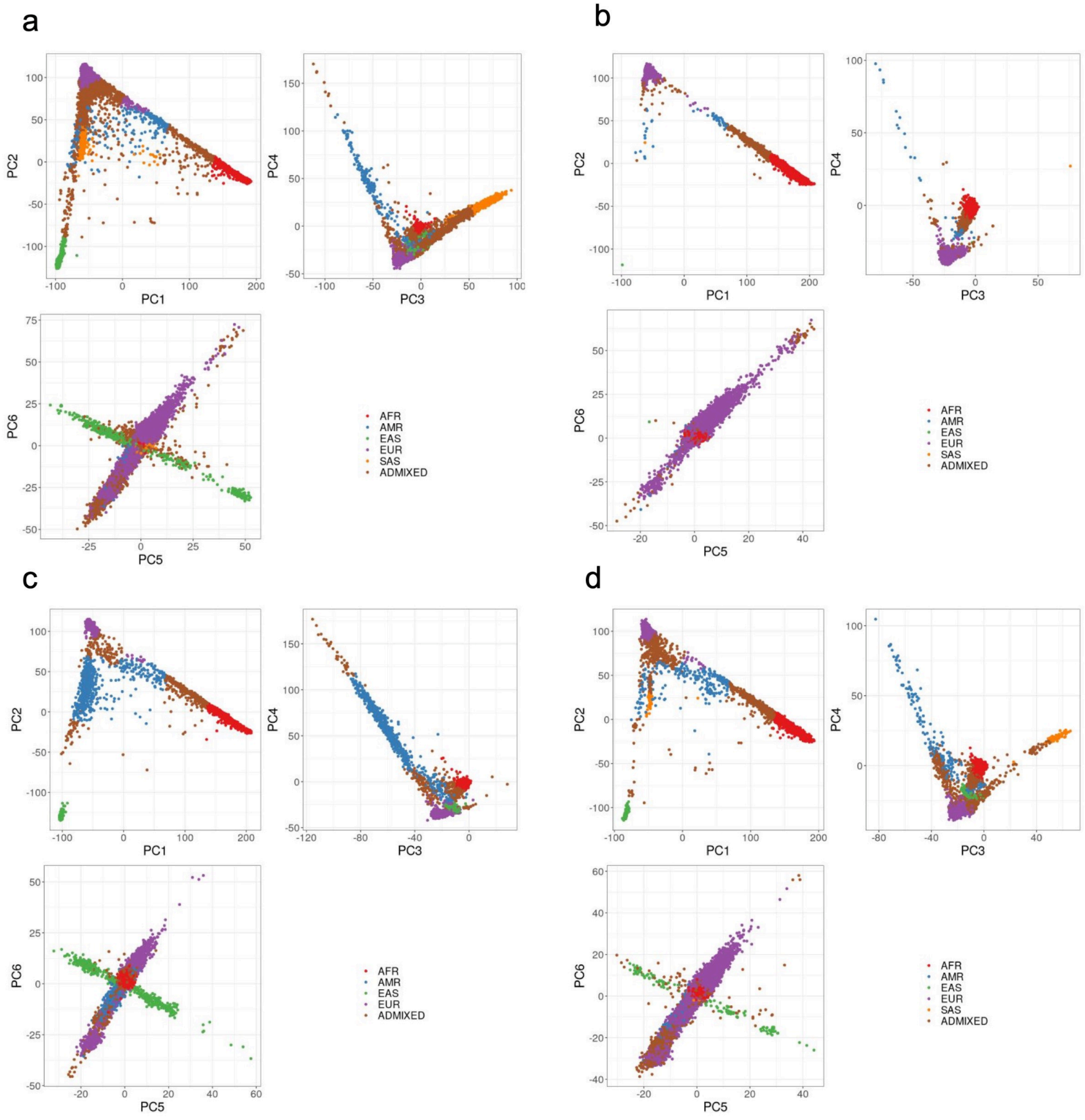

STROBE Statement—Checklist of items that should be included in reports of ***cohort studies***

	Item No	Recommendation	Page No
**Title and abstract**	1	(*a*) Indicate the study's design with a commonly used term in the title or the abstract	1
		(*b*) Provide in the abstract an informative and balanced summary of what was done and what was found	1
**Introduction**			
Background/rationale	2	Explain the scientific background and rationale for the investigation being reported	2-3
Objectives	3	State specific objectives, including any prespecified hypotheses	3
**Methods**			
Study design	4	Present key elements of study design early in the paper	4
Setting	5	Describe the setting, locations, and relevant dates, including periods of recruitment, exposure, follow-up, and data collection	14-18
Participants	6	(*a*) Give the eligibility criteria, and the sources and methods of selection of participants. Describe methods of follow-up<br>(*b*) For matched studies, give matching criteria and number of exposed and unexposed	18-19
Variables	7	Clearly define all outcomes, exposures, predictors, potential confounders, and effect modifiers. Give diagnostic criteria, if applicable	22-25
Data sources/ measurement	8*	For each variable of interest, give sources of data and details of methods of assessment (measurement). Describe comparability of assessment methods if there is more than one group	20-22
Bias	9	Describe any efforts to address potential sources of bias	14
Study size	10	Explain how the study size was arrived at	18
Quantitative variables	11	Explain how quantitative variables were handled in the analyses. If applicable, describe which groupings were chosen and why	21-26
Statistical methods	12	(*a*) Describe all statistical methods, including those used to control for confounding<br>(*b*) Describe any methods used to examine subgroups and interactions<br>(*c*) Explain how missing data were addressed<br>(*d*) If applicable, explain how loss to follow-up was addressed<br>(*e*) Describe any sensitivity analyses	26-27
**Results**			
Participants	13*	(a) Report numbers of individuals at each stage of study—eg numbers potentially eligible, examined for eligibility, confirmed eligible, included in the study, completing follow-up, and analysed<br>(b) Give reasons for non-participation at each stage<br>(c) Consider use of a flow diagram	4, Fig3

Descriptive data	14*	(a) Give characteristics of study participants (eg demographic, clinical, social) and information on exposures and potential confounders<br>(b) Indicate number of participants with missing data for each variable of interest<br>(c) Summarise follow-up time (eg, average and total amount)	Table 1 & 3, sTable3
Outcome data	15*	Report numbers of outcome events or summary measures over time	Table 1 & 3

Main results	16	(*a*) Give unadjusted estimates and, if applicable, confounder-adjusted estimates and their precision (eg, 95% confidence interval). Make clear which confounders were adjusted for and why they were included<br>(*b*) Report category boundaries when continuous variables were categorized<br>(*c*) If relevant, consider translating estimates of relative risk into absolute risk for a meaningful time period	4-5, Fig1, 5-8, Fig2&3
Other analyses	17	Report other analyses done—eg analyses of subgroups and interactions, and sensitivity analyses	sTab2
**Discussion**			
Key results	18	Summarise key results with reference to study objectives	8-9
Limitations	19	Discuss limitations of the study, taking into account sources of potential bias or imprecision. Discuss both direction and magnitude of any potential bias	12-13
Interpretation	20	Give a cautious overall interpretation of results considering objectives, limitations, multiplicity of analyses, results from similar studies, and other relevant evidence	9-11
Generalisabilit y	21	Discuss the generalisability (external validity) of the study results	12-13
**Other information**			
Funding	22	Give the source of funding and the role of the funders for the present study and, if applicable, for the original study on which the present article is based	29,32

*Give information separately for exposed and unexposed groups.

Note: An Explanation and Elaboration article discusses each checklist item and gives methodological background and published examples of transparent reporting. The STROBE checklist is best used in conjunction with this article (freely available on the Web sites of PLoS Medicine at http://www.plosmedicine.org/, Annals of Internal Medicine at http://www.annals.org/, and Epidemiology at http://www.epidem.com/). Information on the STROBE Initiative is available at http://www.strobe-statement.org.
